# A Molecular Phylogeny of the Chalcidoidea (Hymenoptera)

**DOI:** 10.1371/journal.pone.0027023

**Published:** 2011-11-03

**Authors:** James B. Munro, John M. Heraty, Roger A. Burks, David Hawks, Jason Mottern, Astrid Cruaud, Jean-Yves Rasplus, Petr Jansta

**Affiliations:** 1 Department of Entomology, University of California Riverside, Riverside, California, United States of America; 2 INRA, Centre de Biologie et de Gestion des Populations, Montferrier-sur-Lez, France; 3 Department of Zoology, Faculty of Science, Charles University, Prague, Czech Republic; American Museum of Natural History, United States of America

## Abstract

Chalcidoidea (Hymenoptera) are extremely diverse with more than 23,000 species described and over 500,000 species estimated to exist. This is the first comprehensive phylogenetic analysis of the superfamily based on a molecular analysis of 18S and 28S ribosomal gene regions for 19 families, 72 subfamilies, 343 genera and 649 species. The 56 outgroups are comprised of Ceraphronoidea and most proctotrupomorph families, including Mymarommatidae. Data alignment and the impact of ambiguous regions are explored using a secondary structure analysis and automated (MAFFT) alignments of the core and pairing regions and regions of ambiguous alignment. Both likelihood and parsimony approaches are used to analyze the data. Overall there is no impact of alignment method, and few but substantial differences between likelihood and parsimony approaches. Monophyly of Chalcidoidea and a sister group relationship between Mymaridae and the remaining Chalcidoidea is strongly supported in all analyses. Either Mymarommatoidea or Diaprioidea are the sister group of Chalcidoidea depending on the analysis. Likelihood analyses place Rotoitidae as the sister group of the remaining Chalcidoidea after Mymaridae, whereas parsimony nests them within Chalcidoidea. Some traditional family groups are supported as monophyletic (Agaonidae, Eucharitidae, Encyrtidae, Eulophidae, Leucospidae, Mymaridae, Ormyridae, Signiphoridae, Tanaostigmatidae and Trichogrammatidae). Several other families are paraphyletic (Perilampidae) or polyphyletic (Aphelinidae, Chalcididae, Eupelmidae, Eurytomidae, Pteromalidae, Tetracampidae and Torymidae). Evolutionary scenarios discussed for Chalcidoidea include the evolution of phytophagy, egg parasitism, sternorrhynchan parasitism, hypermetamorphic development and heteronomy.

## Introduction

Chalcidoidea (Hymenoptera) are minute wasps that generally range in size from 1-4 mm, with the smallest only 0.11 mm and the largest up to 45 mm. With an estimated diversity of up to 500,000 morphologically distinct species and an even larger number of cryptic species possible [1], [2], [3], [4], this superfamily is likely the most diverse group of insects. While several families are phytophagous (e.g. all Agaonidae; some Eurytomidae, Eulophidae, Pteromalidae, Tanaostigmatidae and Torymidae), most chalcid wasps are parasitoids. They attack immature and adult stages of virtually all insect orders, but have their greatest diversification on the Hemiptera and Holometabola. Because the individual host is killed as a result of parasitoid development, many chalcid species are successfully used as biological control agents of agricultural and ornamental pests (e.g. Aphelinidae and Encyrtidae) [3]. Both economically and ecologically Chalcidoidea have tremendous importance in both natural and managed ecosystems.

Despite their importance, our understanding of their taxonomy and evolutionary relationships is clearly wanting. Partly because of their small size, they are difficult to collect and study, and only about 23,000 species have been described [4]. Nineteen families are currently recognized, with their diversity spread across as many as 80-89 subfamilies, in many cases without consensus on their higher-level placement.

Chalcidoidea and their proposed sister group Mymarommatoidea first appear in mid Cretaceous amber deposits (Mymaridae) [5], [6], [7]. Most extant lineages do not appear until the Eocene, suggesting an extremely rapid post-Cretaceous radiation [6]. However, the presence of Eulophidae and Trichogrammatidae in Late Cenomanian amber from Ethiopia pushes chalcidoid diversification back to the mid Cretaceous, about 93–95 Mya [8].

Synapomorphies uniting most of the members of Chalcidoidea include an exposed prepectus, positioning of the mesothoracic spiracle on the lateral margin of the mesoscutum, wing venation reduced to submarginal, marginal, stigmal, and postmarginal veins, and the presence of multiporous plate sensilla on one or more of the antennal flagellomeres [9], [10]. Molecular evidence places Chalcidoidea as a monophyletic group nested within a monophyletic Proctotrupomorpha and as the sister group to either Diaprioidea or Mymarommatoidea [11], [12], [13], but see Sharanowski et al. [14] for an alternate proposal for Ceraphronoidea as the sister group.

Both morphological and molecular evidence place Mymaridae as the sister group of the rest of Chalcidoidea [10], [11], [13]. A few intuitive hypotheses of relationships within the superfamily have been proposed based on limited morphological justification [5], [15], [16]. However, for relationships within Chalcidoidea, there has not been a morphology-based cladistic analysis across more than just a few inclusive families [9]. A few molecular analyses have addressed relationships broadly across the superfamily, but these have used relatively few taxa to represent such a diverse group [17], [18].

Herein we present the first comprehensive phylogenetic analysis of relationships within the Chalcidoidea using 18S rDNA and the 28S rDNA D2–D5 expansion regions sampled across 722 taxa. The diversity of the superfamily is addressed by the inclusion of 72 subfamilies and 343 genera. Data were aligned according to a secondary structural model, which allows for the unambiguous partitioning of data into conserved regions and regions of ambiguous alignment [19], [20], [21]. Different optimizations of the alignment using MAFFT [22] are analyzed to compensate for potential alignment artifacts and increase phylogenetic resolution. Our analysis provides a new framework for evaluating the composition and relationships of major groups and hopefully will lead to a better understanding of their evolution.

## Materials and Methods

### Taxonomic sampling and specimen vouchering

Sequences were obtained for 722 taxa, with 56 outgroups and 666 ingroups (Table S1). Chalcidoidea are represented by all 19 families, 72 subfamilies, 343 genera and 649 species. Most species are represented by a single specimen; however, to remove any doubt of sequencing error, additional individuals of some species that were difficult to place within any expected grouping (e.g., *Idioporus, Cynipencyrtus* and *Diplesiostigma*) were sequenced. Outgroup taxa included exemplars of Ceraphronoidea (Ceraphronidae and Megaspilidae), Cynipoidea (Cynipidae, Figitidae, Ibaliidae and Liopteridae), Diaprioidea (Diapriidae, Maamingidae and Monomachidae), Mymarommatoidea (Mymarommatidae), Platygastroidea (Platygastridae) and Proctotrupoidea (Heloridae, Pelecinidae, Proctotrupidae, Roproniidae and Vanhorniidae). In the present manuscript we follow the family and subfamily classification of Chalcidoidea of Noyes [4], with additional resolution from the following: Agaonidae follows Cruaud et al. [23], Aphelinidae follows Hayat [24], Chalcididae follows Bouček and Delvare [25] and Narendran [26]; Cleonyminae follows Gibson [27], Eucharitidae follows Heraty [28], Eulophidae follows Burks et al. [29]; Pteromalidae follows Bouček [30], Delucchi [31], Graham [32] and Hedqvist [33], Toryminae follows Grissell [34], and Trichogrammatidae follows Owen et al. [35].

The majority of taxa were sequenced and vouchered at the University of California Riverside (UCR). Additional sequences were provided by co-authors (AC and JYR: Agaonidae and some Pteromalidae; PJ: Torymidae), the HymAToL project (various outgroup taxa), Matt Yoder (NC State University; various outgroup taxa), and Andy Austin (University of Adelaide; various outgroup taxa). See Table S1 for a complete listing of contributed sequences and voucher locations. Taxa sequenced at UCR are represented by either a primary (remains of actual specimen sequenced) or secondary (compared specimen from same collection series) specimen voucher. UCR voucher specimens were each assigned a unique UCRC_ENT Museum identification number and barcode. Additional voucher information is housed in a FileMaker Pro database at UCR developed by JM, and is available on request. UCR vouchers were imaged using a GT-Vision automontage system, with images deposited on MorphBank 4.0 (http://www.morphbank.net/).

### DNA Extraction, Amplification and Sequencing

Genomic DNA extraction at UCR followed a modified version of the Chelex® protocol [36]. Primer sequences for PCR amplification of 18S rDNA and the 28S rDNA D2, D3 and D4+D5 expansion regions are provided in Table 1. Herein, the amplified regions shall be referred to simply as 18Sa-c, D2, D3 and D4+D5. In some cases, a shorter version of 18Sb was amplified with internal primers (18Si, Table 1). Amplification and sequencing followed established protocols at UCR [37]. UCR sequencing was conducted at the San Diego State University Microchemical Core Facility or the UCR Genomics Core Facility. Protocols for the Rasplus lab sequences follow Cruaud et al. [23]. Sequence verification was conducted by comparing forward and reverse sequences. All sequences are deposited on Genbank (Table S1).

**Table 1:** Primer sequences.

Primer Name	Primer Sequence	Reference
28S D2-3551 F	5′ - CGT GTT GCT TGA TAG TGC AGC - 3′	[17]
28S D3-4046 F	5′ - GAC CCG TCT TGA AAC ACG GA - 3′	[134]
28S D2-4057 R	5′ - TCA AGA CGG GTC CTG AAA GT - 3′	[37]
28S D3-4413 R	5′ - TCG GAA GGA ACC AGC TAC TA - 3′	[134]
28S D5-4625 R	5′ - CCC ACA GCG CCA GTT CTG CTT ACC - 3′	[135]
18Sa-1 F	5′ - TAC CTG GTT GAT CCT GCC AGT AG - 3′	[135]
18Sb-441 F	5′- AAA TTA CCC ACT CCC GGC A -3′	[11]
18Sa-591 R	5′- G AAT TAC CGC GGC TGC TGG -3′	[135]
18Si-673 F	5′- ATC GCT CGC GAT GTT TAA CT -3′	[11]
18Si-905 R	5′- AGA ACC GAG GTC CTA TTC CA -3′	[11]
18Sc-1204 F	5′ - ATG GTT GCA AAG CTG AAA C - 3′	[135]
18Sb-1299 R	5′- TGG TGA GGT TTC CCG TGT T - 3′	[11]
18Sc-1991 R	5′ - GAT CCT TCC GCA GGT TCA CCT AC - 3′	[135]

28S primers are named for the relative structural position of the primer (next expansion region in direction of primer), for 18S and 28S their complementary 5′ start position in *D. melanogaster* [131], [132], [133], and whether designated as a forward (F) or reverse (R) primer.

### Secondary structure alignment

Sequences were manually aligned using secondary structure models following Deans et al. [38] and Gillespie et al. [20], [21], [39], [40]. The 18Sa fragment began three bases (TAC) prior to the core helix H9 and included the variable regions V1 and V2 and ended with helix H39'. Fragment 18Sb began four bases (AUAA) prior to the core helix H406a (CGAUACGGGACUC), and included the variable regions V3, V4 (expansion region E23-1 through E23-14) and V5, and ended with core helix H960', just prior to V6. 18Sc began with a conserved loop (AAACCTCA), which preceded H984 and ended with the conserved loop (TGA) between H1506 and H1506', and included regions V6–V9. Amplification of the 28S rDNA D2, D3 and D4+D5 expansion regions began a single base (C) prior to helix H375 (GGGUUGC) in the core region preceding D2 and terminated 2 bases following helix H976 (UGG), subsequent to D5. The final alignment contained 545 blocks of data, which accounted for base-pairing helices and their prime, ambiguously-pairing regions of expansion and contraction (REC), ambiguously-pairing regions of slipped-strand compensation (RSC), non-pairing yet highly conserved loops, and non-pairing and variable loop regions of ambiguous alignment (RAA). For the purposes of this paper, we treat all three of these regions together as RAA regions.

### Comparison between secondary structure and algorithmically generated alignments

Two important aspects of the dataset led us to compare the results obtained with various alignment strategies. First, we are confident of the alignment in the conserved stem-based and core regions; however vagaries of the secondary structure model lead to some local alignments that might not be optimal based on exact pairing of compensatory base changes. Second, distribution and size of RAAs are variable across Chalcidoidea. For such a large matrix, by-eye alignment of these highly-variable ambiguous regions from distantly related taxa is hard to justify. However, these RAAs can be locally informative [11], [29] and we prefer not to exclude them from our analyses. To test different optimizations of our secondary structure alignment and the impact of RAAs, we created two submatrices: one including the conserved stem-based and core regions and another including the regions of ambiguous alignment.

The core secondary structure-derived (SS) submatrix was created by manually removing regions of ambiguous alignment (RAAs), leaving only the structurally aligned helices, core regions, and conserved blocks. As alluded to previously, not all loops are ‘highly variable’ and conserved non-pairing regions, including some loops found in the core, were retained in the SS submatrix.

The second submatrix (RAAs) included the regions of ambiguous alignment *sensu lato* (RAAs, REC, RSCs, and unnamed blocks). An initial 77 regions of ambiguous alignment were identified. Where RECs and their pairing primes bounded an RAA, the blocks were concatenated. Additionally, REC 4 H3q, RAA 24 loop 9, REC 4' H3q', and RAA 25 were concatenated into a single block. Concatenation reduced the number of isolated RAA regions from 77 to 55. Each of these regions was aligned independently and re-included in the corresponding gene region for each of the following datasets.

Sixteen datasets were constructed from these submatrices (Table 2) that can be grouped into four categories: 1) SS submatrix without RAAs; 2–7) SS combined with algorithm-aligned RAAs; 8–10) algorithm-aligned SS submatrix without RAAs; 11–13) algorithm-aligned SS submatrix and algorithm-aligned RAAs, and 14–16) algorithm-aligned dataset in which the SS and RAA submatrices were not treated separately, but with each of the 6 gene regions individually isolated and independently algorithm-aligned.

**Table 2:** Alignment strategies for use of secondary structure and MAFFT alignments of both core/stem (SS) and ambiguous (RAA) regions.

dataset	core/stem	RAA	length	inform.	uninfo.	18Sa	18Sb	18Sc	28S	28S	28S	RAxML	No. of steps
	alignment	alignment							D2	D3	D4-5	best score	SSME data
SSNR	SS	no RAA	2996	853	356	500	757	633	591	333	182	-85277.62	32461
SSGE	SS	guide tree+E-INS-i	4369	1675	566	507	969	701	1302	519	371	-144234.60	32236
SSGL	SS	guide tree+L-INS-i	4369	1676	565	507	969	701	1302	519	371	-144255.37	32223
SSGG	SS	guide tree+G-INS-i	4536	1773	550	507	963	697	1451	531	387	-144123.77	32220
SSME	SS	no guide+E-INS-i	3917	1408	483	506	906	693	993	450	369	-150220.93	31951
SSML	SS	no guide+L-INS-i	3917	1408	487	506	906	693	993	450	369	-150223.77	31957
SSMG	SS	no guide+G-INS-i	3906	1433	468	506	906	694	1023	450	327	-147954.87	31951
MENR	E-INS-i	no RAA	3024	861	375	507	758	634	605	337	183	-85889.86	32522
MLNR	L-INS-i	no RAA	3024	861	374	507	758	634	605	337	183	-85852.51	32483
MGNR	G-INS-i	no RAA	3025	859	380	507	758	634	606	337	183	-85953.75	32527
MEME	E-INS-i	no guide+E-INS-i	3944	1415	502	513	907	694	1007	453	370	-150774.64	32247
MLML	L-INS-i	no guide+L-INS-i	3944	1415	501	513	907	694	1007	453	370	-150775.39	32236
MGMG	G-INS-i	no guide+G-INS-i	3934	1438	492	513	907	695	1038	453	328	-148553.26	32254
MESR	E-INS-i (all data by partition)		4133	1536	553	506	901	693	1196	531	306	-145056.78	31983
MLSR	L-INS-i (all data by partition)		4099	1507	545	506	901	693	1162	531	306	-145084.06	32187
MGSR	G-INS-i (all data by partition)		4139	1519	551	506	901	694	1201	531	306	-145293.59	31997

The guide tree was generated from a RAxML analysis of the SSNR dataset (no RAA). Except for the all data alignments (no submatrix partition), each of the 55 RAA blocks were aligned independently and reinserted into the appropriate gene partition for analysis. E-INS-i, G-INS-i and L-LINS-i are MAFFT alignment options. The RAxML best score was obtained from 10 independent runs using CIPRES v.2.0. The number of informative and uninformative sites and parsimony steps were calculated in PAUP 4.0* for each resulting tree using the SSME dataset.

Automated alignments were performed with MAFFT [22], [41], [42]. Both the online server (v.6) and the downloadable program (v.6.244b) were used to create initial alignments that utilized the following MAFFT algorithms: E-INS-i, G-INS-i and L-INS-i. Alignments for each partition (core region and each of the 55 regions of ambiguous alignment taken independently) were generated using the default settings (gap opening penalty  =  1.53 and offset value  =  0.00).

The RAAs were aligned both with and without a guide tree that was generated using the SSNR (core with no RAA) dataset. Our purpose for using a guide tree was to optimize local alignments for each of the RAAs within terminal clusters of independently recognized taxa grouped through analysis of the SSNR, thus aligning nearest neighbors, as opposed to aligning disparate taxa across the entire dataset without any prior grouping. Maximum likelihood (ML) analyses of this dataset were conducted with RAxML v.7.2.7 using a partitioned GTR+Γ model [43] on the Teragrid cluster, Abe [44] via the CIPRES portal V2.2 [45]. We used 1000 rapid bootstrap (BS) replicates for each run, with initial tests using the autoMRE criterion [46] showing 350 BS to be adequate. A GTRCAT approximation of models was used for ML bootstrapping [47]. Ten RAxML analyses utilizing different starting seeds were executed, followed by ML optimization to find the best-scoring tree. The 10 resulting trees were used to generate a strict consensus tree that was converted to a MAFFT-readable guide tree with the script newick2mafft.rb (http://mafft.cbrc.jp/alignment/software/treein.html). This guide tree was implemented in the MAFFT alignments of the isolated RAAs utilizing the E-INS-i, G-INS-i and L-INS-i algorithms (SSGE, SSGG and SSGL, Table 2).

The secondary structure-derived matrix with MAFFT-aligned RAA regions (SSME) is deposited on Texas A&M's Parasitic Hymenoptera Research Labs' jRNA Secondary Structure and its Phylogenetic Implications website (available through http://hymenoptera.tamu.edu/rna/) and as Supplemental Nexus File S1. The 15 remaining datasets, with and without RAA regions, are available from JMH upon request.

### Dataset partitioning

Sequences were partitioned into six gene regions 18Sa, 18Sb, 18Sc, D2, D3, and D4+D5, with each partition including their respective aligned RAA regions. The 18Sa-c partitions were defined simply as the region sequenced, inclusive of the primers used. The 28S rDNA expansion regions are also contiguous, being bounded on either side by core sequence, which was amplified in the PCR reaction. The decision as to where to define the end of D2 and start of D3 and likewise, the end of D3 and start of D4+D5, was arbitrarily made to fall within the core regions between the expansion regions. The helix H1a' (UUUCAGG), was assigned to mark the end of D2; while the un-named, non-pairing block of sequence (AC), which follows helix H1a' and proceeds helix H563 (CCGU) marked the start of D3. Helix H812 (CCCUCC) was assigned to mark the end of D3, while the un-named, non-pairing block of sequence (GAAG), which follows helix H812 and precedes helix H822 (UUUCC), marks the start of D4+D5.

### Phylogenetic analyses

Maximum Likelihood (ML) analyses and associated bootstrapping (BS) were conducted on the 16 datasets with RAxML v.7.2.7 using a partitioned GTR+Γ model [43] on the Teragrid cluster, Abe [44] via the CIPRES portal V2.2 [45]. A GTRCAT approximation of models was used for ML bootstrapping [47]. To accommodate parameter variation in separate runs [48], 10 analyses were conducted using different seed numbers and 1000 rapid bootstrap (BS) replicates, with the tree with the best known likelihood (BKL) score chosen from among these sets. For comparison of alignments strategies, we examined the number of parsimony informative and uninformative sites, overall length, and the number of step changes mapped with PAUP 4.0* [49] onto each tree using the SSME dataset. The SSME dataset was chosen for the Parsimony analysis, because it provided what we considered to be the optimal results in terms of clade retention and used both the SS and RAA submatrices.

The parsimony analysis of the SSME dataset was conducted with TNT v.1.1 [50], [51]. Heuristic searches were performed using a New Technology Search with default settings, except for using a sectorial search, ratchet weighting probability of 5% with 50 iterations, tree-drifting of 50 cycles, tree-fusing of 5 rounds, and best score hit of 10 times, followed by swapping to completion on all trees found. Nodal supports were calculated using 1000 standard bootstrap replicates.

To be consistent with our interpretation of bootstrap percentage (BP), we use the following scale: a bootstrap percentage of ≥90% is considered very strong, 80–89% means strong, 70–79% means moderate, and 50–70% means low bootstrap support.

To better track relationships, each taxon includes a prefix which is an abbreviation of it family-group (c.f. Table 3, S1), and the suffix includes the DNA voucher code and letters correponding to the gene regions sequenced, corresponding to the three regions of 18S (tuv), 28S-D2 (x), D3 (y) and D4-5 (z).

**Table 3:** Summary of traditional clades within Chalcidoidea, diversity sampled, and support from various datasets and analyses.

						core only		core and RAA				RAxML	TNT
Code	Taxonomy			gen	spp	SSNR	MENR	SSGE	SSME	MGMG	MGSR	MJR*	SSME
AG	**Agaonidae (76/757)**			19	104	100	100	100	100	100	100	100	97
AGA		‘Agaoninae’^a^		12	48	–	–	–	–	–	–	–	–
AG4		‘Agaonidae group 4′		2	3	–	*par*	70	75	86	92	75	–
AGB		‘Blastophaginae’		3	24	–	–	–	–	–	–	–	–
AGK		Kradibiinae		2	25	–	*par*	–	–	–	–	–	–
AGT		Tetrapusinae		1	4	100	100	100	100	100	100	100	100
AP	**Aphelinidae (33/1168)**			21	87	–	–	–	–	–	–	–	–
API		Aphelinidae *incertae sedis*		4	4	n/a	n/a	n/a	n/a	n/a	n/a	n/a	n/a
APA		Aphelininae		7	22	88^b^	88^b^	97^b^	96^b^	91^b^	86^b^	100^b^	56^b^
APAY			Aphytini	3	12	*par*	*par*	*par*	53	*par*	*par*	*par*	+
APZ		Azotinae		1	12	99	100	100	100	100	100	100	99
APC		Coccophaginae		6	43	+	+	81	+	+	+	94	–
APCP			Pteroptricini	5	31	par	par	par	par	par	par	par	–
APE		Eretmocerinae		1	5	100	100	100	100	100	100	100	100
APR		Euryischiinae		2	2	100	100	100	89	100	100	100	100
CAL	**Calesinae (1/4)**			1	3	100	100	100	100	100	100	100	100
CH	**Chalcididae (87/1464)**			20	37	–	–	–	–	–	–	–	–
CHC		Chalcidinae		8	19	–	–	–	–	–	–	–	–
CHCB			Brachymeriini	1	6	100	100	100	100	100	100	100	100
CHCC			Chalcidini	2	8	100	100	100	100	100	100	100	100
CHCR			Cratocentrini	3	3	–	–	–	–	–	–	–	–
CHCP			Phasgonophorini	2	2	98	100	100	100	100	99	100	100
CHD		Dirhininae		1	5	100	100	100	100	100	100	100	100
CHE		Epitranininae		1	3	+	90	99	95	94	98	100	56
CHH		Haltichellinae		8	12	88	90	100	98	98	97	100	+
CHHA			Haltichellini	5	9	+	+	+	*par*	–	56	+	–
CHHY			Hybothoracini	3	3	*par*	*par*	*par*	93	–	*par*	*par*	*par*
CHS		Smicromorphinae		1	1	n/a	n/a	n/a	n/a	n/a	n/a	n/a	n/a
EN	**Encyrtidae (460/3735)**			12	14	+	50	81	72	73	78	100	+
ENE		Encyrtinae		8	9	*par*	*par*	*par*	+	72	+	89	+
ENT		Tetracneminae		4	5	72	69	87	77	97	par	65	+
EU	**Eucharitidae (55/423)**			22	46	100^c^	100^c^	100^c^	100^c^	100^c^	100^c^	100^c^	100^c^
EUE		Eucharitinae		16	27	100	100	100	100	100	100	100	96
EUG		Gollumiellinae		2	3	80	93	98	76	86	99	100	par
EUO		Oraseminae		4	16	par	+	71	+	+	+	75	+
EL	**Eulophidae (297/4472)**			27	28	89^d^	92^d^	99^d^	98^d^	97^d^	98^d^	100^d^	+^d^
ELI		Eulophidae *i.s.*		1	1	n/a	n/a	n/a	n/a	n/a	n/a	n/a	n/a
ELE		Entedoninae		8	8	–	+	50	+	74	59	88	+
ELN		Entiinae		5	6	–	–	67	*par*	+	58	81	+
ELU		Eulophinae		9	10	66	+	96	95	91	85	100	–
ELO		Opheliminae		1	1	n/a	n/a	n/a	n/a	n/a	n/a	n/a	n/a
ELT		Tetrastichinae		3	3	98	98	100	100	100	100	100	99
EP	**Eupelmidae (45/907)**			19	25	–	–	–	–	–	–	–	–
EPC		Calosotinae		5	7	–	–	–	–	–	–	–	–
EPE		Eupelminae		12	14	+	+	+	–	+	–	–	–
EPN		Neanastatinae		2	4	–	–	–	+	–	–	–	–
EY	**Eurytomidae (88/1424)**			14	28	–	–	–	–	–	–	–	–
EYE		Eurytominae		9	14	100^e^	99e	100^e^	100^e^	100^e^	100^e^	100^e^	100^e^
EYH		Heimbrinae		1	1	n/a	n/a	n/a	n/a	n/a	n/a	n/a	n/a
EYR		Rileyinae		2	7	+	+	97	90	87	87	100	+
LEU	**Leucospidae (4/134)**			2	6	98	90	100	100	98	98	100	98
MY	**Mymaridae (103/1424)**			13	15	98	95	100	99	98	97	100	61
MYI		Mymaridae *i.s.*		1	1	n/a	n/a	n/a	n/a	n/a	n/a	n/a	n/a
MYA		Alaptinae		3	3	–	–	–	–	–	–	–	–
MYE		Eubronchinae		1	2	99	100	98	99	100	87	100	84
MYM		Mymarinae		8	9	–	–	–	–	–	–	–	–
ORM	**Ormyridae (3/125)**			2	3	66	56	67	+	61	52	100	+
PE	**Perilampidae (15/277)**			14	34	+^f^	+^f^	–	–	–	–	–	–
PEI		Perilampidae *i.s.*		1	1	n/a	n/a	n/a	n/a	n/a	n/a	n/a	n/a
PEA		Akapalinae		1	1	n/a	n/a	n/a	n/a	n/a	n/a	n/a	n/a
PEM		Philomidinae		3	3	99	98	100	100	100	100	100	97
PEC		Chrysolampinae		4	9	73	67	88	72	68	80	100	–
PEP		Perilampinae		5	20	96	98	100	100	100	99	100	76
PT	**Pteromalidae (588/3506)**			111	130	–	–	–	–	–	–	–	–
PTI		Pteromalidae *i.s.*		2	2	n/a	n/a	n/a	n/a	n/a	n/a	n/a	n/a
PT01		Asaphinae		3	3	–	–	–	–	–	–	+	–
PT02		Ceinae		1	2	93	93	100	98	98	99	100	98
PT03		Cerocephalinae		3	3	99	99	100	100	100	100	100	100
PT04		Chromeurytominae		1	1	n/a	n/a	n/a	n/a	n/a	n/a	n/a	n/a
PT05		Cleonyminae		10	10	–	–	–	–	–	–	–	–
PT05D			Chalcedectini	1	1	n/a	n/a	n/a	n/a	n/a	n/a	n/a	n/a
PT05C			Cleonymini	3	3	68	56	84	54	+	52	100	+
PT05L			Lyciscini	5	5	+	+	92	55	+	+	100	+
PT05O			Ooderini	1	1	n/a	n/a	n/a	n/a	n/a	n/a	n/a	n/a
PT06		Coelocybinae		4	4	–	–	–	–	–	–	–	–
PT07		Colotrechninae		2	2	–	–	–	–	–	–	–	–
PT08		Cratominae		1	1	n/a	n/a	n/a	n/a	n/a	n/a	n/a	n/a
PT09		Diparinae		6	8	–	–	–	–	–	–	–	–
PT09D			Diparini	4	4	–	–	–	–	–	–	–	–
PT09N			Neapterolelapini	1	2	57	55	96	73	63	+	81	–
PT10		Epichrysomallinae		16	28	100	100	100	100	100	100	100	93
PT11		Eunotinae		6	7	–	–	–	–	–	–	–	–
PT11E			Eunotini	4	5	52^g^	75^g^	90^g^	86^g^	93^g^	98^g^	100^g^	61^g^
PT11M			Moranilini	1	1	n/a	n/a	n/a	n/a	n/a	n/a	n/a	n/a
PT11T			Tomocerodini	1	1	n/a	n/a	n/a	n/a	n/a	n/a	n/a	n/a
PT12		Eutrichosomatinae		1	1	n/a	n/a	n/a	n/a	n/a	n/a	n/a	n/a
PT13		Herbertiinae		1	1	n/a	n/a	n/a	n/a	n/a	n/a	n/a	n/a
PT14		Leptofoeninae		2	3	–	–	–	–	–	–	–	–
PT15		Macromesinae		1	1	n/a	n/a	n/a	n/a	n/a	n/a	n/a	n/a
PT16		Miscogasterinae		9	10	–	–	–	–	–	–	–	–
PT16M			Miscogasterini	5	6	–	–	–	–	–	–	–	–
PT16S			Sphegigasterini	2	2	–	–	–	–	–	–	–	–
PT16T			Trigonoderini	2	2	–	–	–	–	–	–	–	–
PT17		Ormocerinae		6	5	–	–	–	–	–	–	–	–
PT17M			Melanosomellini	3	3	–	–	*par*	–	+	–	–	–
PT17S			Systasini	1	1	n/a	n/a	n/a	n/a	n/a	n/a	n/a	n/a
PT18		Otitesellinae		3	4	par	–	–	–	–	–	–	–
PT19		Panstenoninae		1	2	96	89	98	98	84	77	100	96
PT20		Pireninae		4	4	–	–	–	–	–	–	–	–
PT21		Pteromalinae		17	18	–	–	–	–	–	–	–	–
PT21P			Pteromalini	4	4	–	–	–	–	–	–	*par*	–
PT22		Spalangiinae		1	3	100	100	100	100	100	100	100	100
PT23		Sycoecinae		1	1	n/a	n/a	n/a	n/a	n/a	n/a	n/a	n/a
PT24		Sycophaginae		5	6	82	94	91	81	77	91	100	+
PT25		Sycoryctinae		2	2	–	–	–	–	–	–	–	–
ROT	**Rotoitidae (2/2)**			1	1	n/a	n/a	n/a	n/a	n/a	n/a	n/a	n/a
SI	**Signiphoridae (4/76)**			8	26	81	80	95	98	97	97	100	52
SIS		Signiphorinae		1	9	100	100	100	100	100	100	100	99
SIT		Thysaninae		3	12	*par*	*par*	*par*	*par*	*par*	*par*	*par*	*par*
TAN	**Tanaostigmatidae (9/92)**			4	5	98^h^	95^h^	99^h^	100^h^	99^h^	100^h^	100^h^	77^h^
TE	**Tetracampidae (15/50)**			6	7	–	–	–	–	–	–	–	
TEM		Mongolocampinae		1	1	n/a	n/a	n/a	n/a	n/a	n/a	n/a	n/a
TEP		Platynocheilinae		1	1	n/a	n/a	n/a	n/a	n/a	n/a	n/a	n/a
TET		Tetracampinae		4	5	100^i^	100^i^	100^i^	100^i^	100^i^	100^i^	100^i^	97^i^
TO	**Torymidae (68/986)**			29	41	–	–	–	–	–	–	–	–
TOM		Megastigminae		3	6	66	67	99	99	97	97	100	92
TOT		Toryminae		28	37	–	+	67	+	+	62	86	+
TOTI		Toryminae *i.s.*		3	4	n/a	n/a	n/a	n/a	n/a	n/a	n/a	n/a
TOTM			Microdonteromerini	6	8	–	–	–	*par*	*par*	*–*	*par*	*par*
TOTN			Monodontomerini	6	8	80	*par*	100	91	89	81	100	97
TOTP			Palachiini	2	2	–	–	–	–	–	–	–	–
TOTO			Podagrionini	4	4	*par*	57	*par*	90	*par*	55	62	+
TOTT			Torymini	3	6	75	74	66	87	68	66	100	–
TOTY			Torymoidini	4	5	*par*	–	–	–	–	–	88	–
TR	**Trichogrammatidae (83/839)**			12	21	–	+	61	65	64	+	94	+
TRO		Oligositinae		9	10	98	100	97	96	95	93	100	+
TROI		Oligositinae *i.s.*		3	4	n/a	n/a	n/a	n/a	n/a	n/a	n/a	n/a
TROC			Chaeotostrichini	2	3	99	100	100	100	100	100	100	100
TROO			Oligositini	1	2	100	100	100	100	100	100	100	100
TROP			Paracentrobiini	1	1	n/a	n/a	n/a	n/a	n/a	n/a	n/a	n/a
TRT		Trichogrammatinae		3	11	+	*par*	*par*	*par*	*par*	*par*	*par*	*par*
TRTI		Trichogrammatinae *i.s.*		3	5	n/a	n/a	n/a	n/a	n/a	n/a	n/a	n/a
TRTT			Trichogrammatini	2	6	100	100	100	100	100	100	100	100
**Number of clades with positive support:**						**56**	**59**	**60**	**58**	**58**	**59**	**62**	**52**

Dataset abbreviations explained in Table 4. RAxML majority rule (MJR) is a consensus across all 16 submatrices. Support values are bootstrap percentages. The number of clades with positive support is summed for all clades with either a *+* (presence) or numerical support; *par*  =  paraphyletic; –  =  not monophyletic. Estimated diversity (genera/species) after family group names from Noyes [4]. Taxa represented by a single OTU or *incertae sedis* (*i.s*.) were considered not applicable (n/a) for clade support.

a =  without Agaonidae Group 4 (*Wiebesia* and *Blastophaga* R1757);

b =  without Azotinae or *Eretmocerus*;

c =  excluding Akapalinae and Philomidinae;

d =  without *Trisecodes*;

e =  excluding *Buresium*;

f =  including *Idioporus*;

g =  excluding *Idioporus*;

h =  not including *Cynipencyrtus*;

i =  excluding *Diplesiostigma*.

## Results

### Alignment models, tree length and clade support

Summaries of the 16 datasets generated from the two submatrices are presented in Table 2. The core region (SS) was 2996 bp in length and only slightly shorter than the MAFFT alignment of the same data (3,024–3,025 bp), with the differences accumulated mostly in the 28S D2 region. The application of the guide tree to the RAAs produced the longest alignment (4,369–4,536 bp) with the greatest impact on the length of the 28S D2 and D3 regions. Application of the guide tree greatly increased the number of parsimony informative sites (1,675–1,773 bp), the number of uninformative (autapomorphic) sites (550–565 bp), and had the greatest impact on tree length using the SSME dataset as a metric (32,220–32,236 steps) (Table 2). The MAFFT aligned RAAs without a guide tree were added to both the core region (SSME, SSMG and SSML) and to the MAFFT alignment of the core region (MEME, MGMG and MLML). Using mapped state changes and the SSME metric, the core + no guide tree RAAs datasets produced the shortest tree topologies (31,951–31,957 steps). Both the alignment length, and the RAxML best score differed very little within the different MAFFT variants of each alignment model. The MAFFT alignment of all data without regard to partition (MESR, MGSR and MLSR) produced an alignment of intermediate length (4,099–4,139 bp).

#### Phylogenetic Analyses

**Figure 1:**
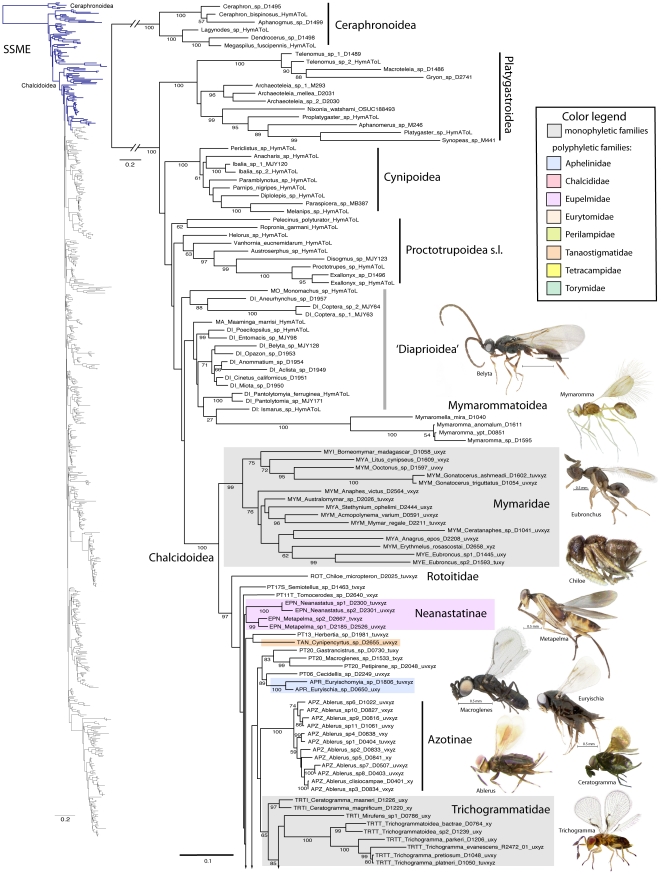
Phylogenetic tree from secondary structure alignment of stem data and E-INS-i alignment of RAAs (3917 aligned; SSME). RAxML analysis with seed 38652 and 1000 rbs bootstrap replicates (support >50% above branches). Phylogram of entire tree on left colored to match inset. Taxon names with prefix indicating classification (see Table 3) and suffix indicating DNA voucher number and gene regions included for 18Sa-c (tuv) and D2 (x), D3 (y) and D4-5 (z). Monophyletic families indicated by gray shading; polyphyletic families other than Pteromalidae indicated according to inset color scheme.

A summary of supported clades across six of the 16 analyses is presented in Tables 3 and 4, along with a summary of the >50% majority rule consensus support (MJR) across all 16 best known likelihood (BKL) RAxML trees. We present the BKL tree from the SSME RAxML result (Figs 1–7), with the caveat that this represents only one summary of relationships found within Chalcidoidea. The clade support tables are a better representation of the support for traditional subfamily and family groups (Table 3) and for some higher-level relationships (Table 4). When present, bootstrap support on Figures 1–7 generally corresponds with support across all analyses. Surprisingly, there was little impact of alignment strategy (SS or MAFFT) on the results, except for a slight increase in support for various clades at all levels with the inclusion of RAAs (core and RAA, Tables 3, 4).

**Figure 2 pone-0027023-g002:**
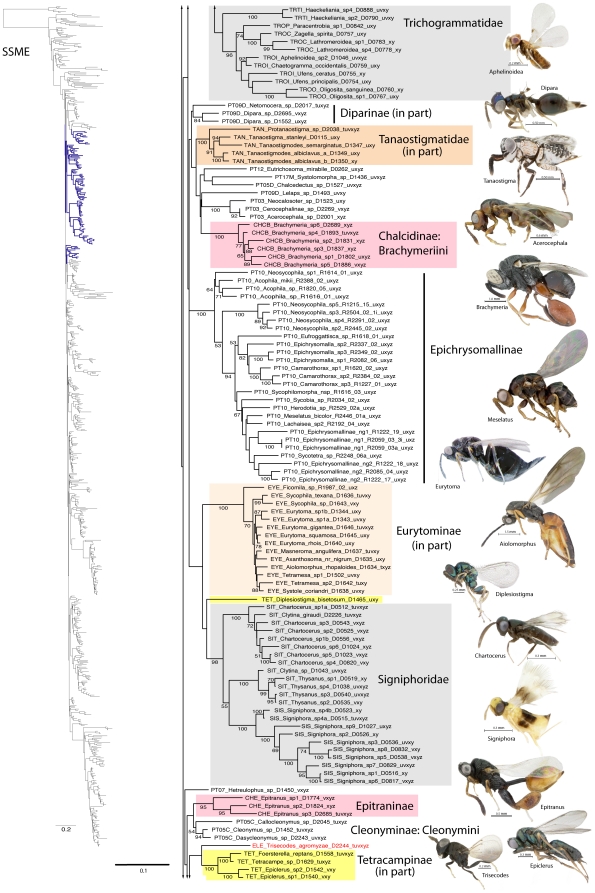
Phylogenetic tree of Chalcidoidea (continued).

**Figure 3 pone-0027023-g003:**
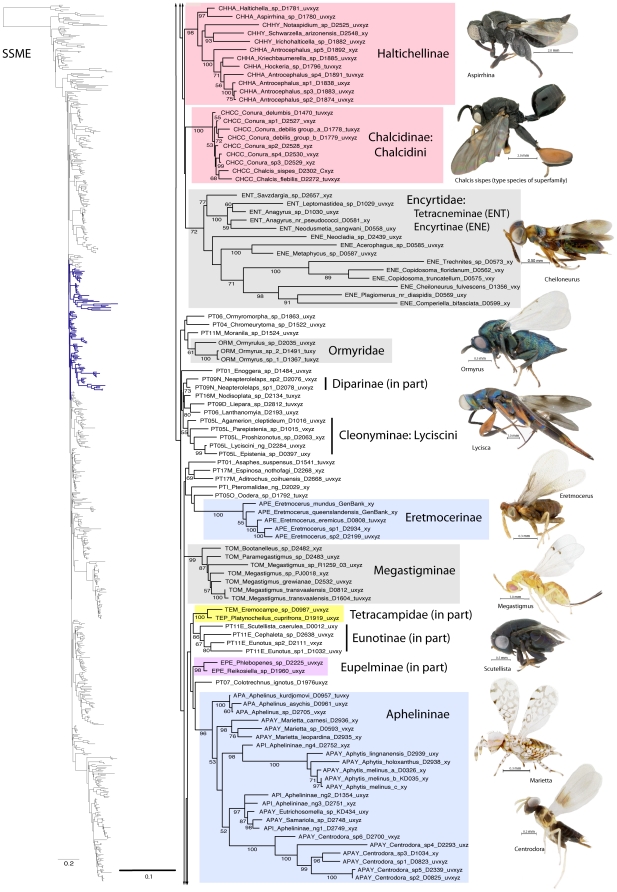
Phylogenetic tree of Chalcidoidea (continued).

**Figure 4 pone-0027023-g004:**
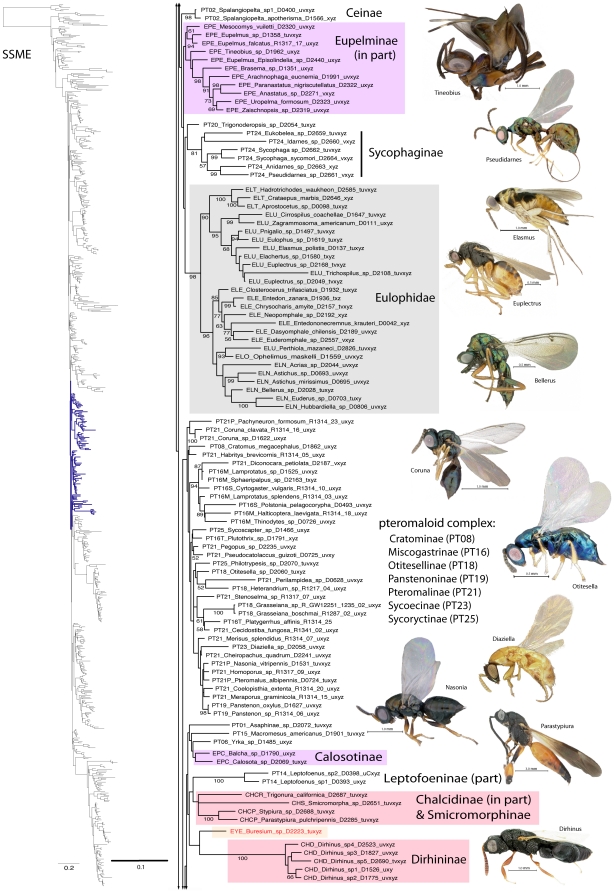
Phylogenetic tree of Chalcidoidea (continued).

**Figure 5 pone-0027023-g005:**
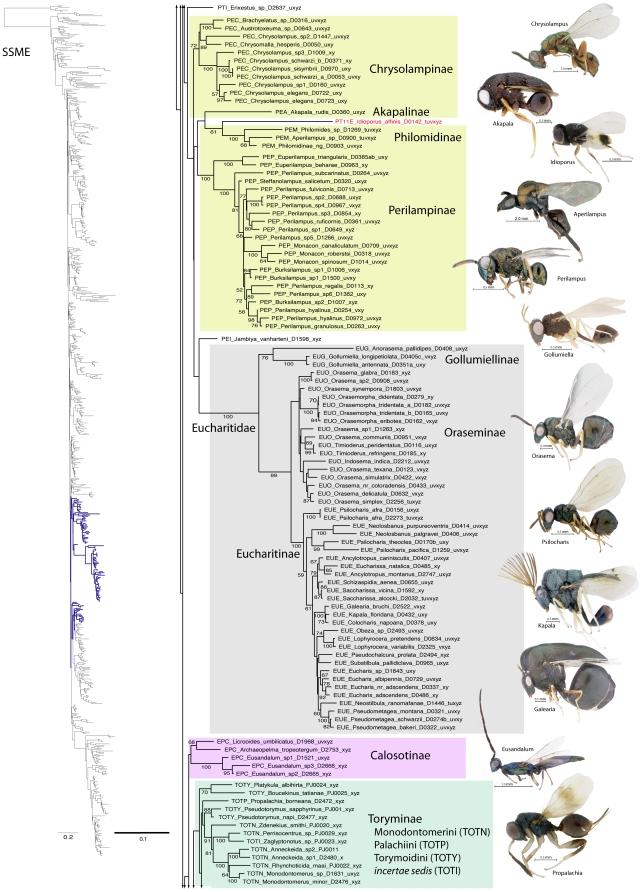
Phylogenetic tree of Chalcidoidea (continued).

**Figure 6 pone-0027023-g006:**
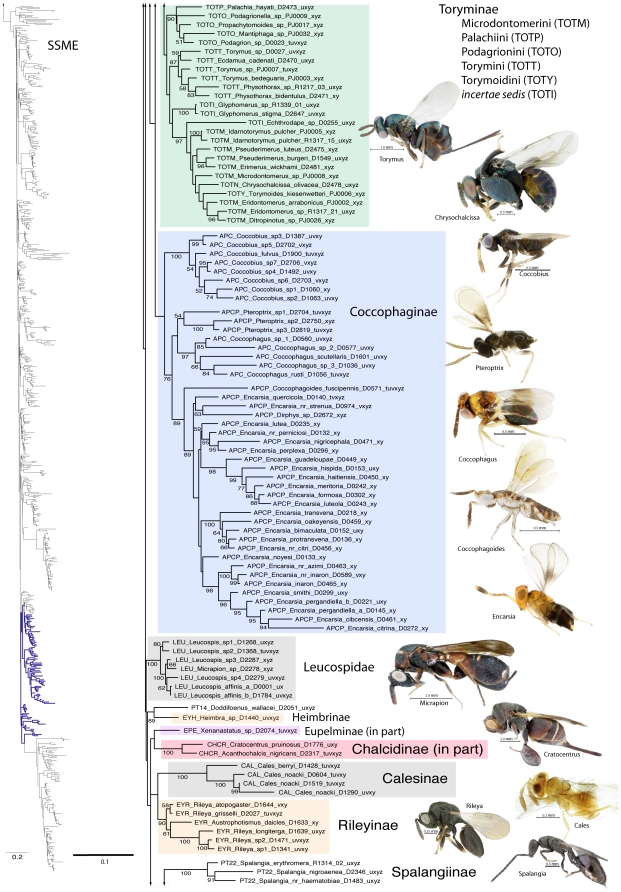
Phylogenetic tree of Chalcidoidea (continued).

**Figure 7:**
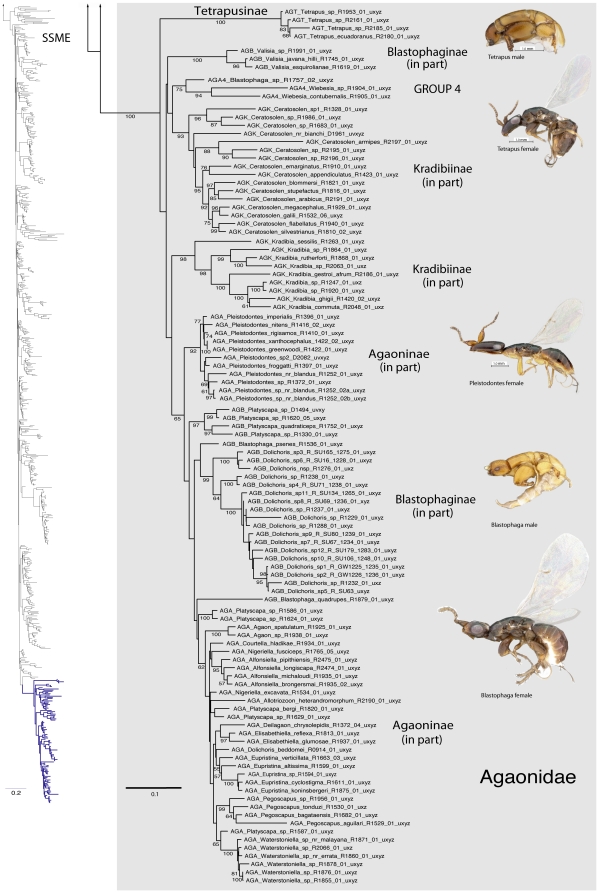
Phylogenetic tree of Chalcidoidea (continued).

**Table 4:** Higher group relationships supported across various analyses.

	core only		core and RAA				RAxML	TNT
Group Relationships	SSNR	MENR	SSME	SSGE	MGSR	MGMG	MJR	SSME
*Pantolytomiya* + Chalcidoidea	−	+	−	+	−	+	62	−
Diaprioidea (part) + Chalcidoidea	−	−	+^a^	−	−	−	56	−
‘Diapriidae’ + Chalcidoidea	+	−	−	−	−	−	−	−
Mymarommatoidea + Chalcidoidea	−	−	−	−	+	−	−	−
(Proctotrupoidea + Diaprioidea) sister to Chalcidoidea	−	−	−	−	−	−	−	+
Chalcidoidea	99	95	100	100	98	98	100	100
remaining Chalcidoidea minus Mymaridae	91	55	97	95	55	85	94	+
remaining Chalcidoidea minus Rotoitidae and Mymaridae	+	+	+	76	+	+	94	−
Mymaridae: 4−segmented taxa	74	78	75	87	57	80	88	+
Mymaridae: 5-segmented taxa	+	+	76	62	83	+	88	+
Eulophidae: (Opheliminae + *Perthiola*) + Entiinae	−	−	+	+	−	−	56	+
Eucharitidae + Perilampidae	−	−	+	+	+	+	−	+
Perilampidae (with Akapalinae, Philomidinae and *Idioporus*)	+	+	*par*	+	+	+	−	−
*Jambiya* + Eucharitidae	−	−	+	+	+	+	−	+
*Jambiya* + Perilampidae	−	+	−	−	−	−	−	−
pteromaloid complex^b^	+	+	+	+	+	+	−	+^c^
Spalangiinae + Agaonidae	−	−	+	−	−	−	−	−
Sycophaginae + Agaonidae	+	−	−	−	−	−	−	−
remaining Agaonidae minus Tetrapusinae	+	55	+	−	−	+	−	+
Aphelininae + Coccophaginae	+^d^	−	−	−	−	−	−	−
Azotinae + Trichogrammatidae	+	+	+	−	+	+	62	−
Azotinae + Signiphoridae	−	−	−	−	−	−	−	+
Agaoninae + Blastophaginae (excluding group 4)	+	+	65	61	+	+	62	+

a =  Monomachidae + Diapriidae as sister groups;

b =  includes Cratominae, Miscogastrinae, Otitesellinae, Panstenoninae, Pteromalinae and Sycoryctinae;

c =  without *Heterandrium* (Otitesellinae);

d =  including *Platygerrhus* (Microgasterinae: Trigonoderini).

Dataset abbreviations explained in Table 4. RAxML majority rule (MJR) is a consensus across all 16 submatrices. Support values are bootstrap percentages. Abbreviations: + refers to presence of clade but without numerical support; *par*  =  paraphyletic.

Interestingly, the automated (MAFFT) alignments of all data were comparable in clade support to any of the divided alignment strategies based on recognizing the core and stem data. There was slightly better clade support using G-INS-i when applied to data that included RAAs.

### Informativeness of RAAs

**Figure 8:**
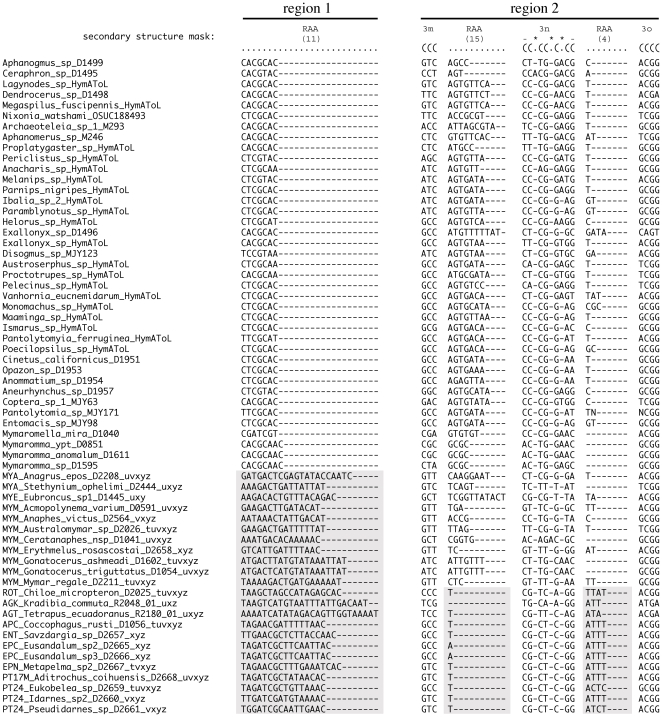
Examples of structural support from two sections of 28S-D2 (indicated by bar) for outgroups and a sampling of Chalcidoidea. RAA(11) shows an increase in the number of nucleotides and a decrease in the degree of conservation for Chalcidoidea including Mymaridae (highlighted). In all Chalcidoidea excluding Mymaridae, RAA(15) undergoes a dramatic decrease to either 1 or no nucleotides and RAA(4) shows a slight increase in size. The bordering alignment around RAA(15) demonstrates compensatory changes in helices 3m, 3n and 3o.

Within 28S and 18S, distinct structural differences occur between RAA regions for the outgroups, Mymaridae, and the remaining Chalcidoidea taxa. For example, RAA(11) shows a pattern of increase in the number of bases and an associated decrease in degree of conservation for Chalcidoidea in comparison to the outgroup taxa (Fig. 8). Alternatively, RAA(15) reduces to a single nucleotide for Chalcidoidea, with the exclusion of Mymaridae. Within the same region, RAA(4) shows a slight but more subtle increase for Chalcidoidea excluding Mymaridae. RSC(4) and RSC(4′) both show support for Chalcidoidea excluding Mymaridae based on a respective increase to a 4 base motif (RSC 4), and an increase to a consistent AT or GT pattern (RSC 4′; not shown). These structural changes support both monophyly of Chalcidoidea and a sister group relationship between Mymaridae and the remaining Chalcidoidea. No RAA patterns were observed that would add support for relationships in the outgroup taxa. However within Chalcidoidea, additional structural changes within variable regions add support to some relationships (i.e., an increase in 18S loop(4) size in Perilampidae and Eucharitidae; and deletion of a contiguous variable region (RAAs 23-25) in Eulophinae + Tetrastichinae). Six variable regions in Agaonidae demonstrate substantial growth in size, both across and within the family, that distinguish them from all other Chalcidoidea. The different sizes of the variable regions might be expected to have the greatest impact on results from datasets contrasting the inclusion or exclusion of RAAs, or the MAFFT alignment without reference to the SS core structure; however, overall there appeared to be no impact, with all results consistently supporting monophyly of Chalcidoidea and a sister group relationship between Mymaridae and the remaining Chalcidoidea.

Inclusion of the RAAs contributed to the monophyly of Encyrtinae, Entedoninae and Entiinae (Table 3). Their inclusion increased the BS support for a number of clades, including Agaoninae group 4, Encyrtidae, Eulophinae, Rileyinae, Lyciscini, Eunotini, Signiphoridae and Megastigminae (Tables 3, 4). At a higher group level, the inclusion of the RAA regions provided a greater amount of support for Eucharitidae + Perilampidae, and the genus *Jambiya* as the sister group of Eucharitidae. In no cases did the inclusion of RAAs result in a substantial decrease in support for a clade.

### Phylogenetic Relationships

Relationships across the 16 ML analyses overall were the same regardless of alignment method or the inclusion or exclusion of RAAs (Figs. 1–7, Tables 3, 4). The parsimony analysis of the SSME dataset produced more than 10,000 most parsimonious trees of 31,607 steps (RI = 0.62); however the strict consensus was well resolved (Supplementary Fig. S1) and in general accord with the likelihood results.

Outgroup relationships generally favored a paraphyletic Diaprioidea as sister group to Chalcidoidea (Fig. 1), but in a few cases Mymarommatoidea were the proposed sister group. A core Proctotrupomorpha clade of Proctotrupoidea *sensu stricto*, Diaprioidea, Mymarommatoidea and Chalcidoidea were supported in all results. Both Ceraphronoidea and Platygastroidea were distantly related in all analyses.

Chalcidoidea were always monophyletic with strong support, as was a sister group relationship between Mymaridae and the remaining Chalcidoidea (Table 4). *Chiloe micropteron* (Rotoitidae) was consistently supported in the likelihood results as the sister group of the remaining Chalcidoidea excluding Mymaridae (94% MJR), but with bootstrap support only in the SSGE results (BS 76). However, in the parsimony results *Chiloe* was deeply nested within Chalcidoidea (Supplementary Fig. S1).

Relationships within Chalcidoidea were highly variable along the backbone of the tree and should be regarded as a broad polytomy, but with consistent and sometimes strong support for many traditional taxon groupings at the family, subfamily, and tribe levels (Table 3). There is sometimes a lack of support for families that can be defined by several justifiable synapomorphies such as Chalcididae, and there is consistent support for some other families such as Eulophidae that are founded on what might be considered as weak loss or reductive features [9].

## Discussion

### Comparison of alignment strategies

Overall, there was little impact of the application of different MAFFT alignments to either the RAA regions, the core secondary structure data, or to the different gene regions without reference to secondary structure. This is optimistic for the future inclusion of new taxa to our data set where we can avoid the labor-intensive approach of having to align new taxa to our existing secondary structure model. Inclusion of the RAAs contributed to monophyly and clade support for a number of taxa, and also increased support at higher levels. Furthermore, structural differences found in various RAAs (Fig. 8) provide clear support for Chalcidoidea, a sister-group relationship between Mymaridae and other Chalcidoidea, and for some of the higher-level groups within Chalcidoidea. Clearly, RAAs do provide some phylogenetic signal and their inclusion in analyses is warranted despite some authors recommending complete [52] or partial [19] deleting of these regions.

### Outgroup relationships

We found either Mymarommatoidea or Diaprioidea as the sister group of Chalcidoidea. These equivocal results were similar to results from a recent analysis of Hymenoptera that used more extensive molecular data from four gene regions and nearly complete 28S and 18S data [11]. Molecular data from both studies clearly support a monophyletic group of Diaprioidea, Mymarommatoidea and Chalcidoidea within the Proctotrupomorpha. With the inclusion of morphological data in a combined analysis, Mymarommatoidea is the sister group of Chalcidoidea [13], as hypothesized by Gibson [10]. Unfortunately, the biology of Mymarommatoidea remains unknown, making it difficult to compare with Chalcidoidea.

### Phylogenetic relationships within Chalcidoidea

Chalcidoidea are well supported as monophyletic. Mymaridae are strongly supported as monophyletic and the sister group of the remaining Chalcidoidea. This hypothesis was first proposed by Gibson [10] based on morphology, and substantiated by Heraty et al. [11] and Sharkey et al. [13]. *Chiloe micropteron* (Rotoitidae) was the sister group of the remaining Chalcidoidea in all of the likelihood results, but not using parsimony. With more extensive gene sampling, Heraty et al. [11] recovered the same relationships in likelihood analyses of the eye-aligned data, and with parsimony only in the data aligned by eye. Mymaridae and Mymarommatidae are both common in early to mid Cretaceous amber deposits [5], [6], [8], which support their early origin and sister group relationships. Rotoitidae is unknown in any fossil deposits, but has a potentially archaic pattern of distribution, with genera known only in New Zealand and southern Chile [6], suggesting a late cretaceous origin [53].

After Rotoitidae, the relationships within Chalcidoidea become vague. The backbone of the chalcidoid tree has little support, with taxonomic groups shifting in different analyses from the base to somewhere more apical in the topology. As well, there are few consistent sister group relationships supported among the higher-level groups. One of the few relationships that can be substantiated based on larval morphology, Eucharitidae + Perilampidae [54], occurs in some but not all results, and never has bootstrap support. This is not simply an artifact of our ribosomal dataset; similar results with poor backbone support were also found by Desjardins et al. [18] using 4 nuclear protein coding genes and far fewer taxa. We do recover support for many of the traditional higher-level groups within Chalcidoidea, mostly at the subfamily and tribe level, but also for a few diverse family groups such as Agaonidae, Eulophidae, Eucharitidae and Trichogrammatidae. We also recovered consistent support for a novel pteromaloid complex that is a mix of morphologically very distinct subfamily groups. For some of the traditionally well-supported groups such as Chalcididae, the majority of the included taxa were monophyletic in only one analysis. A similar rare grouping was also found for a monophyletic Signiphoridae + Azotinae.

We found some taxa that could not be placed within any traditional higher-level group. There were also a few singleton taxa that defied placement, including *Diplesiostigma*, *Cynipencyrtus* and *Idioporus*. Interestingly, *Idioporus* is also difficult to place based on morphology, although neither Perilampidae (likelihood) or Rotoitidae (parsimony) were ever suggested as being related based on a morphological study by LaSalle et al. [55]. Calesinae are currently *incertae sedis* within Chalcidoidea [56], and our results to not offer any potential sister groups for this clade. Pteromalidae, as expected, is polyphyletic and affects greatly the composition and relationships of other taxa. Our results will be reevaluated in a combined morphological analysis, which is currently underway (Heraty et al. in prep), but it is clear that the family level relationships of Chalcidoidea are in need of major revision.

For the discussions below, some historical information on relationships is presented for each family group followed by the results of the current study. A more detailed review of classification history and biology can be found in Gibson et al. [9] and Hanson & Gauld [57]. We try not to discuss relationships of taxa within supported clades, but most often species within the same genera and species groups were monophyletic, and relationships within a clade were generally the same across different analyses (Figs 1–7).

#### Agaonidae

Agaoninae and Sycophaginae (as Idarninae), once included in Torymidae, were moved to Agaonidae by Bouček [30]. Agaonidae *sensu lato* were comprised of Agaoninae, Epichrysomallinae, Otitesellinae, Sycoecinae, Sycophaginae and Sycoryctinae [58]. Bouček noted that there were no unique morphological characters to define Agaonidae *sensu lato*, yet argued against limiting the family to the pollinating group (Agaoninae) and suggested a sister-group relationship of at least Agaoninae + Sycophaginae. Grissell [34] suggested that Agaonidae (*sensu lato*) may form a derived clade within the Torymidae. Rasplus et al. [59] revised the Agaonidae, having determined that it was not monophyletic, limiting the family to include only Agaoninae (Agaonidae *sensu stricto*). Cruaud et al. [23] analyzed relationships within Agaonidae *s.s.* and proposed up to four subfamilies, Tetrapusinae, Agaoninae group 4 (potential subfamily), ‘Blastophaginae’ and ‘Agaoninae’, but with the latter two groups likely collapsing into a single subfamily Agaoninae.

Agaonidae (*sensu stricto*) was monophyletic in all analyses with likelihood BS values of 100% and parsimony support of 97%. Tetrapusinae were recovered with 100% BS in all analyses (Table 3), and were either sister group to the remaining Agaoninae, as reported in [23], or nested within Agaonidae (Table 4). Agaonidae Group 4 was monophyletic in all of the likelihood results, but not parsimony. Kradibiinae were never recovered as monophyletic, although both genera, *Kradibia* and *Ceratosolen*, were each monophyletic. Agaoninae were rendered paraphyletic in all analyses by Blastophaginae, but a monophyletic group of Agaonidae + Blastophaginae, excluding Agaonidae Group 4, was recovered in most results with low support (Table 4).

None of the other subfamilies previously placed in Agaonidae were placed near to Agaonidae, although in the SSNR dataset (core only), Sycophaginae were placed as the sister group of Agaonidae but without bootstrap support.

#### Aphelinidae

Woolley [60] suggested that monophyly of Aphelinidae was not certain, and noted the historical tendency to group all parasitoids of adult and nymphal Hemiptera into Aphelinidae without an understanding of relationships. Presently, most authors recognize that Aphelinidae may be paraphyletic if not polyphyletic [9], [17], [61]. Characters uniting the Aphelinidae may also not be apomorphic [24], [62]. Based on only a few taxa, Aphelinidae were paraphyletic in the molecular analysis of Campbell et al. [17]. Previous authors have placed aphelinids within various families, including Eulophidae [63], [64], Encyrtidae [65], [66], Pteromalidae [62] or as a distinct family [67]. Rosen and DeBach [68] noted that Aphelinidae share morphological affinities with both Encyrtidae (shape of the mesopleura and structure of the pro- and mesotibial spurs) and Eulophidae (thoracic sclerite morphology and antennal segmentation). Gibson [69] hypothesized an Aphelinidae + Signiphoridae relationship on the basis of the structure of the mesotrochantinal plate and metasternum, a relationship also proposed by Domenichini [70]. Woolley [71] found strong morphological evidence uniting Azotinae + Signiphoridae. Compere and Annecke [67] and Rosen and DeBach [68] considered Aphelinidae to be more closely related to Signiphoridae and Encyrtidae. Viggiani and Battaglia [72] proposed that Aphelinidae were morphologically allied with Eulophidae and Trichogrammatidae. Relationships within Aphelinidae are just as, if not more, complex [24], [63], [73], [74], [75], [76], [77], [78], [79], [80], [81], [82]. The most recent treatment of Aphelinidae [24] recognizes the following subfamilies and tribes; Aphelininae (tribes Aphelinini, Aphytini, Eretmocerini and Eutrichosomellini), Eriaphytinae, Azotinae, Coccophaginae (tribes Coccophagini, Physcini and Pteroptricini), Eriaporinae and Euryischiinae. Noyes [4] uses Eretmocerinae, which we follow herein. Calesinae were excluded from Aphelinidae by Hayat [24].

Our results lend support to the idea that Aphelinidae are not monophyletic (Figs 1–6). At best, the two subfamilies Aphelininae (excluding *Eretmocerus*) + Coccophaginae were monophyletic in the SSNR analysis. Aphelininae, Azotinae (*Ablerus*), Eretmocerinae (*Eretmocerus*) and Euryischiinae were each recovered with very strong BS support in all analyses (Table 3). Coccophaginae were monophyletic in the majority (94%) of likelihood analyses, but *Coccobius* was excluded from the other taxa in the parsimony results (Table 3). In the majority of cases, the aphelinine tribes Aphelinini (*Aphelinus*), Aphytini, and Eutrichosomellini (all Aphelininae) are monophyletic, although Eutrichosomellini often renders Aphytini paraphyletic. Within Coccophaginae, *Coccophagus* consistently rendered Pteroptricini paraphyletic. Within Pteroptricini, *Encarsia* is consistently rendered paraphyletic by *Dirphys*.

There was no consistent or plausible sister group taxon for Aphelininae or Coccophaginae. In the majority of analyses, Euryischiinae is sister to *Cecidellis* sp. (Coelocybinae: Pteromalidae), which can be justified morphologically (RGB). The monogeneric Eretmocerinae is monophyletic with strong support in all results, but has no association with other aphelinid taxa. Azotinae were always monophyletic, with 100% bootstrap support, with former members of *Azotus* rendering *Ablerus* paraphyletic, which is an expected result. Azotinae were the sister group to Trichogrammatidae in the likelihood results, but without bootstrap support (Table 4). Monophyly of Azotinae + Signiphoridae is supported by several morphological synapomorphies [71], but this group was recovered only in the parsimony results (Table 4).

### Calesinae (unplaced to family)

*Cales* (Calesinae) were excluded from Aphelinidae and left unplaced in Chalcidoidea by Hayat [83]. Mottern et al. [56] recently reviewed the Calesine, and discussed its unique morphology and potential relationships with various taxa, including Aphelinidae, Eretmocerinae, Eulophidae, Mymaridae and Trichogrammatidae.

Calesinae were monophyletic with 100% BS support in all analyses (Fig. 6). Included in our analysis are two morphological and geographically distinct species, *Cales berryi* from New Zealand, and *Cales noacki* from South America, including Chile. This same pattern of distribution was used as an argument for the archaic placement of Rotoitidae. Although *Cales* was intermediate between Mymaridae and other Chalcidoidea in Campbell et al. [17], it was always well nested within Chalcidoidea in all of our results. No consistent outgroups were identified in any of our results.

#### Chalcididae

Bouček and Halstead [84] noted that the classification of Chalcididae has changed little over the years. A sister-group relationship with Leucospidae or even the inclusion of Leucospidae within Chalcididae was suggested by Gibson [16], [85]. Monophyly of Chalcididae has not been previously doubted, largely based on four morphological synapomorphies [86], [87]. Traditional classifications have included Chalcidinae with the tribes, Chalcidini, Cratocentrini, Phasgonophorini and sometimes Brachymeriini, with other subfamilies including Dirhininae, Epitraninae, Haltichellinae and Smicromorphinae [30], [88]. In a phylogenetic analysis of the family, Wijesekara [86] proposed that Smicromorphinae were nested within Chalcidinae, with Chalcidinae including Smicromorphinae sister to the remaining chalcidids, followed by a sequence of Cratocentrinae, Brachymeriinae (Brachymeriini + Phasgonophorini), and finally Dirhininae (Dirhinini + Epitranini) + Haltichellinae (Haltichellini + Hybothoracini). Noyes [4] did not recognize Brachymeriinae, which is the convention followed herein.

Chalcididae were not monophyletic in any of our analyses. The MENR analysis produced the closest approximation to a monophyletic Chalcididae, with a grouping of *Dirhinus* (Dirhininae), *Epitranus* (Epitraninae), Chalcidinae, *Brachymeria* (Brachymeriinae), Phasgonophorini and *Trigonura* (Cratocentrini). However, this group surprisingly also included two pteromalid subfamilies (Macromesinae and Leptofoeninae) and excluded *Cratocentrus* and *Acanthochalcis* (Cratocentrini). Otherwise, the various groups were inconsistent in their grouping in the other analyses. At the subfamily level, Epitraninae, Dirhininae and Haltichellinae were all monophyletic with very strong BS support (Table 3). Smicromorphinae included only a single taxon, and was either independent from other chalcidids or it grouped with Cratocentrini or Phasgonophorini, but never with Chalcidini as proposed by Wijesekara. The subfamily Chalcidinae were never monophyletic, but the tribes Brachymeriini, Chalcidini and Phasgonophorini all had very high BS support across all analyses (Table 3). Interestingly, our Old World representatives of *Chalcis* (the type genus of the superfamily; occurring Worldwide) render the widespread New World genus *Conura* paraphyletic in all analyses. While monophyly of Haltichellinae was supported in all analyses, monophyly of the two tribes, Haltichellini and Hybothoracini, varied.

Our results do not offer much resolution for the relationships within Chalcididae, but do offer support for recognition of Brachymeriinae, Dirhininae, Epitraninae, Chalcidinae (as Chalcidini), Haltichellinae and Smicromorphinae. Both Phasgonophorini and Cratocentrini are less easily placed, and we could not recover the monophyly of the Cratocentrini (*Trigonura* and *Acanthochalcis* + *Cratocentrus*) in any of our analyses. Leucospidae never grouped with any of the chalcidid families, which contradicts hypotheses that they are the sister group of Chalcididae, or that they might render Chalcididae paraphyletic.

#### Encyrtidae

The monophyly of Encyrtidae is not questioned and there is strong morphological evidence to support this family [89]. An Encyrtidae + Tanaostigmatidae sister-group relationship has often been proposed, with this clade in turn being sister to Eupelmidae [69], [89], [90], [91]. Noyes et al. [89] followed the division of Encyrtidae into the subfamilies Tetracneminae and Encyrtinae [92], [93], [94] and noted that while Tetracneminae is undoubtedly monophyletic, Encyrtinae may represent a paraphyletic assemblage.

Encyrtidae were monophyletic across all analyses, with moderate to very strong BS support from the likelihood analyses with RAAs included (Table 3). Tetracneminae were monophyletic with moderate to very strong support across most analyses, with Encyrtinae forming either a paraphyletic or monophyletic sister group. The extraordinary branch lengths found within Encyrtidae (Fig. 3) occur in the results of both SS and SS + RAA analyses, and thus are not simply the result of having several taxa with long RAA inserts. Our results never supported a close relationship with *Cynipencyrtus,* Tanaostigmatidae or any of the eupelmid subfamilies.

#### Eucharitidae

Several morphological features support the monophyly of Eucharitidae [28]. Largely on the basis of the highly sclerotized first instar larva (planidium), Heraty and Darling [54] proposed a sister-group relationship for Eucharitidae and Perilampidae. Based on molecular and morphological evidence, Gollumiellinae form the sister group of Oraseminae + Eucharitinae [6], [37]. Akapalinae and Philomidinae were proposed as belonging to Eucharitidae by Bouček [30]. Philomidinae share planidial larvae with Eucharitidae [95], but immatures of Akapalinae are unknown.

Eucharitidae *sensu stricto* (Gollumiellinae, Oraseminae and Eucharitinae) were monophyletic with 100% BS support across all analyses. Akapalinae were grouped with Perilampinae in all of the likelihood results, but as the sister group of Eucharitidae *s.s.* in the parsimony analysis. Philomidinae were never grouped with Eucharitidae.

While Eucharitinae were always very strongly supported, Oraseminae was occasionally paraphyletic to Eucharitinae. Gollumiellinae was paraphyletic only in the parsimony analysis. Monophyly of Psilocharitini (*Psilocharis* and *Neolosbanus*) is not supported, which is similar to results from other molecular studies [37].

**Figure 9:**
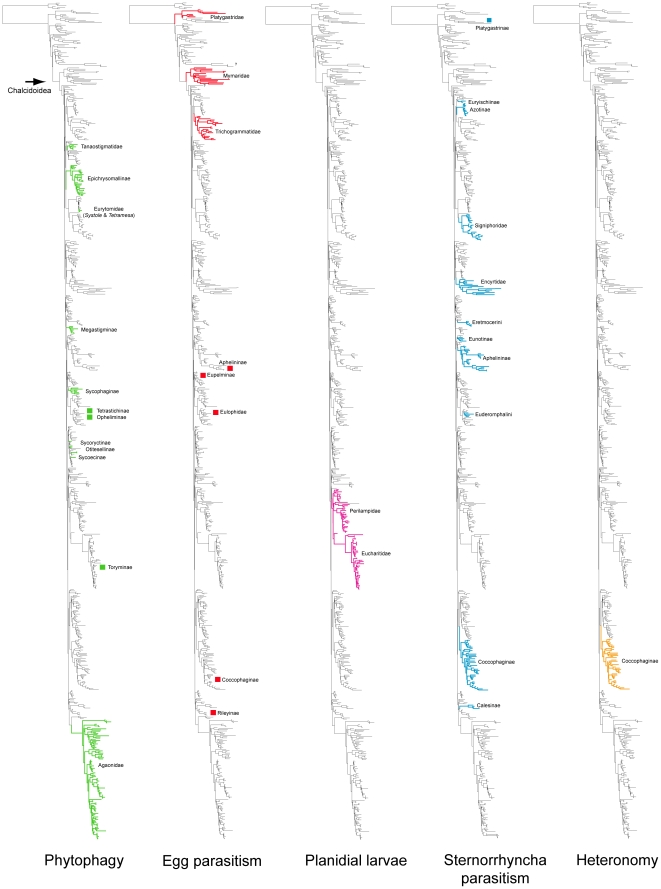
Five life history traits mapped onto SSME likelihood tree. Colored squares refer to presence of a trait in a clade, but not in a member sampled in this study.

A Eucharitidae + Perilampidae sister group was retrieved in most of the likelihood analyses that included RAAs, and also in the parsimony analysis (Table 4); however, without bootstrap support. Morphological support for this group rests on the presence of a sclerotized planidial first-instar larvae [54], [95], and we place some degree of confidence in results that support their monophyly. With the inclusion of Philomidinae in this clade, it would support a single origin of planidia larvae within Chalcidoidea (Fig. 9). However, parsimony results supported a monophyletic Perilampidae + Eucharitidae, without Philomidinae, which was grouped instead with some Phasgonophorini (Chalcididae) and Rileyinae (Eurytomidae).

#### Eulophidae

Monophyly of Eulophidae generally has not been challenged, although morphological support is based almost entirely on character reduction [29]. Based largely on molecular evidence, Elasmidae was synonymized with Eulophidae by Gauthier et al. [96]. At a higher level, Schauff et al. [97] suggested a grouping of Eulophidae, Elasmidae and Trichogrammatidae, but made note that there was no strong evidence for such a relationship. Eulophinae were suggested to be the most basal of the four subfamilies due to their “less-specialized features” [97]. In a combined analysis, Burks et al. [29] proposed that Eulophinae + Tetrastichinae were the sister group of (Opheliminae + Entiinae) + Entedoninae. The only eulophid with three-segmented tarsi, *Trisecodes*, was removed from Entedoninae and placed as *incertae sedis* within Eulophidae [29]. The whitefly parasitoid group Euderomphalini were sister group to Entedonini in Entedoninae, which was contrary to their placement in Entiinae by Gumovsky [98].

Eulophidae were monophyletic with strong to very strong support in all of our analyses (Fig. 4, Table 3), but with the exclusion of *Trisecodes*, which in all analyses was sister group to taxa outside Eulophidae. Support was consistently very high for Tetrastichinae, and increased with the inclusion of RAAs for Entedoninae, Entiinae and Eulophinae. As proposed by Gauthier et al. [96], *Elasus* (formerly Elasmidae) was always nested within Eulophinae. As well, Tetrastichinae and Eulophinae (including *Elasmus*) have a unique deletion of a contiguous variable region (RAAs 23-25). *Perthiola* (Anselmellini) was always the sister group *Ophelimus* with high bootstrap support. Anselmellini were placed outside of Eulophinae by Gauthier et al. [96]. With added resolution from the RAAs, *Perthiola* + Opheliminae grouped either with Entiinae (54% of likelihood trees and parsimony; Table 4) or with Entedoninae. Without the RAAs, these four groups were monophyletic but unresolved. Our results support the hypothesis of relationships suggested by Burks et al. [29], and substantiate the potential inclusion of Anselmellini within Opheliminae.

The exclusion of *Trisecodes* from Eulophidae as proposed by Burks et al. [29] is justified. This genus was usually placed (81% of likelihood analyses and parsimony), but without strong support, as the sister group of Tetracampinae (excluding *Diplesiostigma*), and was never grouped with other Eulophidae.

Importantly, there was no relationship supported for Eulophidae with any of the aphelinid subfamilies, including Calesinae, which have many similar reductive features [56]. The analyses without RAAs (SSNR, MENR) did support a Eulophidae + (Azotinae + Trichogrammatidae) clade, but otherwise there were no consistent outgroups, and never any groups that have been previously proposed in the literature.

#### Eupelmidae

While there is strong morphological support for the monophyly of each of the three subfamilies of Eupelmidae, it has been proposed that the family might represent a grade rather than a clade [9], [69], [99], [100]. The grade was implicated to include Encyrtidae and Tanaostigmatidae, and potentially Aphelinidae, which all share an expanded acropleuron and other associated features; however, there is also a possibility of closer relationships of one or more subfamilies to Cleonyminae (Pteromalidae) [69].

Eupelmidae were never monophyletic. Also, its subfamilies Calosotinae, Eupelminae, and Neanastatinae were almost never monophyletic. The SSME dataset was one of the rare instances in which Neanastatinae were monophyletic (Fig. 1), but in the same results both Calosotinae and Eupelminae occur twice in very different parts of the tree (Figs 3–6). Eupelminae were monophyletic in some analyses, including both datasets that did not include the RAAs (Table 3). Calosotinae were never monophyletic, with *Calosota* and *Balcha* grouping distantly from *Archaeopelma*, *Licrooides* and *Eusandalum*. None of the Eupelmidae ever grouped with Tanaostigmatidae or Encyrtidae.

#### Eurytomidae

The monophyly of Eurytomidae was recently questioned as no synapomorphies defining the family are known [101]. Indeed, the molecular analyses of Campbell et al. [17] and Chen et al. [102] and the morphological analyses of Lotfalizadeh et al. [103] failed to recover a monophyletic Eurytomidae. Stage & Snelling [104] recognized Heimbrinae, Rileyinae and Eurytominae, with the latter including the previously recognized Buresiinae. Chen et al. [102] proposed elevating Rileyinae to family status, while Lotfalizadeh et al. [103] found Rileyinae to consist of two clades of unrelated taxa (*Rileya* and *Macrorileya* + *Buresium*). Both molecular and morphological investigations found *Eurytoma* to be polyphyletic [102], [103].

Eurytomidae was never recovered as monophyletic in any of our analyses. However, Eurytominae (excluding *Buresium*) were monophyletic in all of our analyses with very high support (Table 3). *Rileya* (Rileyinae) were monophyletic in all analyses, but with very high support only in the likelihood analyses when RAAs were included. Both *Heimbra* (Heimbrinae) and *Buresium* (Eurytominae) never grouped with the other eurytomid genera. No logical outgroups were identified.

#### Leucospidae

Leucospidae are generally recognized as a monophyletic group of four genera closely related to Chalcididae [86], [105]. However, characters proposed to support the monophyly of this combined lineage are all problematic and potentially convergent [9], [86].

Leucospidae were monophyletic and had greater than 90% support across all analyses. Our one species of *Micrapion* (South Africa) consistently rendered *Leucospis* (worldwide representation) paraphyletic. No close association with Chalcididae was found.

#### Mymaridae

Although there was some early doubt about the monophyly of Mymaridae [106], the family has been well substantiated based on morphology and molecular evidence [17], [107], [108]. Huber [108] noted that the higher classification of Mymaridae is unstable, and as per the advice of Huber and Triapitsyn (personal communication) Mymaridae subfamilies have been abandoned and genera grouped according to their number of tarsal segments. Gibson [10] was the first to propose morphological evidence that Mymaridae might be the sister group of the remaining Chalcidoidea, but without firm resolution.

Mymaridae were found to be monophyletic in all analyses with very strong support (Fig. 1, Table 3). The 4-segmented tarsi group, represented by the genera *Borneomymar*, *Gonatocerus*, *Litus* and *Ooctonus*, were consistently monophyletic across all analyses with moderate to strong support (Table 4). The remaining genera, *Acmopolynema*, *Anagrus*, *Anaphes*, *Australomymar*, *Ceratanaphes*, *Erythmelus*, *Eubroncus*, *Mymar* and *Stethynium*, formed the 5-segmented tarsi group. This group is supported in most analyses (88% of likelihood analyses), with moderate to strong BS support only when RAAs were included. There was no support for Mymarinae or Alaptinae. Eubronchinae were monophyletic, but these were represented by only a single genus. Mymaridae were strongly supported as the sister group of the remaining Chalcidoidea in all analyses.

#### Ormyridae

Hanson [109], noted that the status and relationships of Ormyridae are uncertain. Members of the family have been included as a subfamily in Pteromalidae [111], Torymidae [112], or as their own family [30].

The two genera, *Ormyrus* and *Ormyrulus*, were monophyletic in all of our analyses but with low to very strong BS support (Fig. 3). In 56% of the likelihood analyses, all based on use of the core SS alignment and with or without RAAs, supported a sister-group relationship with *Moranila* (Pteromalidae: Eunotinae: Moranilini), but otherwise there were no consistent outgroup associations, and never any close association with either of the torymid subfamilies.

#### Perilampidae

The limits of Perilampidae are not clear, with variable inclusion of the subfamilies Chrysolampinae, Philomidinae and Perilampinae, and treatment of each or all groups as a separate family or subfamily of Pteromalidae [9], [100], [113]. *Akapala* (Akapalinae) were initially placed in Perilampidae, but later transferred to Eucharitidae [30]. More recently, *Jambiya* was described and included within Perilampidae, but an association with either Chrysolampinae or Perilampinae could not be made [114]. *Jambiya* has an enlarged ovipositor, which is also a feature of basal lineages of Eucharitidae, and a relationship with that family cannot be rejected. A proposed relationship between Perilampidae, Philomidinae and Eucharitidae is based on presence of a planidial larva [54], [95].

In likelihood results, Perilampidae *sensu stricto* (Chrysolampinae + Perilampinae) was never recovered. With RAAs excluded, a monophyletic ‘Perilampidae’ was recovered with low support that included Chrysolampinae (67-73% BS), Perilampinae (96-98% BS), Akapalinae, Philomidinae and *Jambiya*. This group also included the pteromalid genus *Idioporus* (Pteromalidae: Eunotinae: Eunotini). In these analyses, Eucharitidae and Perilampidae were not monophyletic. With the inclusion of RAAs, the results are more variable, but often recover Perilampidae and Eucharitidae as monophyletic, *Jambiya* as sister group to Eucharitidae, but again with Philomidinae, Akapalinae and *Idioporus* nested within a paraphyletic or monophyletic Perilampidae, but still with Chrysolampinae and Perilampinae each monophyletic (Fig. 5). A monophyletic Perilampidae *s.s.* (Chrysolampinae + Perilampinae) was recovered only in the parsimony analysis. These results also supported *Jambiya* as the sister group of Akapalinae + Eucharitidae. Philomidinae were distantly placed with Phasgonophorini (Chalcididae) and Rileyinae (Eurytomidae). Thus, while Eucharitidae *s.s.* is well supported, there is conflicting support for the definition of Perilampidae and a definitive association with Eucharitidae.

#### Pteromalidae

Pteromalidae are essentially a dumping-ground for presumably monophyletic groups that cannot be assigned to established families and which lack family status in their own right [9]. Herein, we recognize the 30 subfamilies of Noyes [4], as well as the three non-pollinator fig-wasp associated subfamilies assigned to Pteromalidae (Otitesellinae, Sycoecinae and Sycoryctinae) or placed as *incertae sedis* (Epichrysomallinae and Sycophaginae) by Rasplus et al. [59]. Historically, many pteromalid subfamilies were elevated to family status, only to once again resume subfamily status within Pteromalidae [9]. There has been no comprehensive morphological analysis of the family. Molecular analyses have supported the concept of a polyphyletic assemblage, but even the most comprehensive studies have sampled relatively few taxa across the spectrum of the family [17], [18]. We were able to sample 25 of these 36 subfamilies, and where possible sample more extensively within groups (Table 3). We limit our discussion below to significant groupings or results. Notably, many of the taxa are ‘almost’ monophyletic, often with the exclusion of one or more taxa, and many of these cases will need to be evaluated elsewhere.

Pteromalidae were expected to be polyphyletic [9], [15], and were never retrieved as monophyletic. Several subfamilies were monophyletic and very strongly supported across all analyses including Ceinae (*Spalangiopelta*), Cerocephalinae, Epichrysomallinae, Panstenoninae (*Panstenon*), Pteromalinae, Spalangiinae (*Spalangia*) and Sycophaginae. In no case did support increase with the addition of RAAs. Of interest is the a novel grouping of the pteromalid subfamilies Cratominae (*Cratomus*), Miscogastrinae (except *Nodisoplata*), Otitesellinae, Panstenoninae, Pteromalinae, Sycoecinae (*Diaziella*) and Sycoryctinae. This grouping occurs in all analyses, including parsimony, but without bootstrap support. A clade of Miscogastrinae and Pteromalinae was strongly supported by Desjardins et al. [18], but none of these other subfamilies were included as part of that study. This ‘pteromalid complex’ is peculiar for its small amount of molecular divergence and high degree of morphological complexity, especially for the non-pollinating fig wasps Otitesellinae and Sycoryctinae. The low divergence and stability across various analyses suggest that the subfamilies in this group might eventually be synonymized under Pteromalinae. The taxononic placement of *Nodisoplata*, which was placed outside of this complex, needs to be reconsidered. The two other two fig-wasp associated subfamilies, Epichrysomallinae and Sycophaginae, were monophyletic but not associated with any consistent outgroup taxon. In one analysis without RAAs (SSNR), Sycophaginae were the sister group of Agaonidae, but without BS support. This result was proposed by Copland and King [115].

Coelocybinae, Ormocerinae, Pireninae and Pteromalinae were never monophyletic. Cleonyminae were polyphyletic. In all analyses, Cleonymini and Lyciscini were each monophyletic with low support in all analyses, with Lyciscini gaining increased support from the inclusion of RAAs. Chalcedectini (*Chalcedectus*) had variable relationships, but never with other Cleonyminae. Ooderini (*Oodera*) had sister-group relationships that varied from Leucospidae to Encyrtidae, and on two occasions, Lyciscini. Cratominae (*Cratomus*) had variable relationships throughout the analyses, but often occurred in the pteromalid complex as suggested by its morphology. Diparinae were never monophyletic, as also found by Desjardins et al. [18]. Eunotinae were never retrieved as monophyletic, and the tribes Moranilini and Tomocerodini, each represented by a single taxon, were inconsistently allied with other families. Eunotini were monophyletic and strongly supported in all of the analyses. Surprisingly, Leptofoeninae, which have strong morphological support, were never monophyletic. Ormocerinae were never monophyletic. Sycoryctinae and Otitesellinae were consistently polyphyletic which is a result supported by morphology [59]. Within Otitesellinae, the two *Grasseiana* species form a monophyletic group, while *Heterandrium* sp. and *Otitesella* sp. were inconsistently allied with other taxa. Panstenoninae were nested within Pteromalinae. Pireninae and Pteromalinae were never monophyletic. Spalangiinae were always monophyletic, but were never recovered with a consistent sister group.

For Pteromalidae, our results are similar to those of Desjardins et al. [18] based on an analysis of four protein coding genes. The family is polyphyletic with respect to most Chalcidoidea and few of the higher-level assemblages can be consistently grouped with other pteromalid or chalcidoid groups.

#### Rotoitidae

In their description of the family, Bouček and Noyes [116] noted that Rotoitidae may be the sister group of Tetracampidae and Eulophidae. Other potential associations have included Eulophidae, Mymaridae, Trichogrammatidae and Tetracampidae [15], [16]. Based on an analysis of both distribution and ovipositor morphology, Gibson & Huber [117] concluded that Rotoitidae might be the second most ancestral lineage of Chalcidoidea after Mymaridae, but noted that features of the antenna and mesosoma conflict with this conclusion.

Rotoitidae were represented by one species, *Chiloe micropteron*. In all but one of the likelihood analyses, it was basal and sister to the remaining Chalcidoidea after Mymaridae, with BS support for a monophyletic Chalcidoidea after Rotoitidae only in the SSGE results. The alternate likelihood result placed it as the sister group of Mymaridae, thus still basal within the superfamily. Parsimony results have *Chiloe* nested within Chalcidoidea as the sister group of *Idioporus* (Eunotinae: Eunotini) in a clade with *Systolomorpha* (Pteromalidae: Ormocerinae: Melanosomellini) and Trichogrammatidae. No morphological features would support this alternative hypothesis.

#### Signiphoridae

There is little doubt over the monophyly of Signiphoridae; however, Thysaninae may be paraphyletic with respect to Signiphorinae [71], [118]. Gibson [69] suggested a relationship between Signiphoridae and Aphelinidae, or members within Aphelinidae. Woolley [71] proposed a Signiphoridae + Azotinae sister group based on an unsegmented antennal club, presence of an epiproct [70] posterior to the syntergum in all female Azotinae and Signiphoridae, and apodemes projecting forward from the anterolateral angles of sterna 3 to 6 of the metasoma of females. Pedata and Viggiani [119] alluded to an azotine + signiphorid relationship with the discovery of tubercles above the spiracles of third instar *Ablerus perspeciosus* and *Signiphora flavella* larvae.

Signiphoridae and Signiphorinae (*Signiphora*) both monophyletic with very strong support across all analyses (Table 3). Thysaninae were paraphyletic in all of our results. The placement of *Clytina* was puzzling, with *C. giraudi* rendering *Chartocerus* paraphyletic in all analyses, while *Clytina* sp. D1023 was consistently the sister group of *Thysanus*.

Signiphoridae were not placed with Azotinae, or any logical outgroup, in any of the likelihood analyses. In these analyses, Azotinae was consistently the sister group of Trichogrammatidae. However, in the parsimony analysis, Azotinae and Signiphoridae were monophyletic and did not group with Trichogrammatidae.

#### Tanaostigmatidae

Tanaostigmatidae *sensu* LaSalle [90] is a distinct monophyletic group. LaSalle and Noyes [91] transferred *Cynipencyrtus* from Encyrtidae to Tanaostigmatidae, yet noted that this genus was morphologically and biologically distinct from other members of the family. It has been argued that *Cynipencyrtus* could be sister to Encyrtidae, sister to Tanaostigmatidae + Encyrtidae, or sister to Tanaostigmatidae alone [9], [69], [99]. There is strong morphological support for monophyly of the Tanaostigmatidae + Encyrtidae clade, but weaker support for the inclusion of Eupelmidae within this group [9].

Tanaostigmatidae *sensu stricto* (without *Cynipencyrtus*) was always monophyletic with strong support. *Cynipencyrtus* was variously allied with other taxa throughout the different analyses, and tanaostigmatids were never the sister group of Encyrtidae. This disparate grouping may be an artifact of the larger analysis, as we have been able to recover Tanaostigmatidae + (*Cynipencyrtus* + Encyrtidae) in a study with a smaller and more selective sampling of taxa (Mottern & Heraty, unpublished).

#### Tetracampidae

Tetracampidae probably represents a polyphyletic assemblage with three extant subfamilies [120]. There is considerable argumentation for placement of the different subfamilies as Aphelinidae, Eulophidae or Pteromalidae [9], [30], [55].

Tetracampidae were never monophyletic in our analyses. Excluding *Diplesiostigma*, Tetracampinae were monophyletic and very strongly supported. *Diplesiostigma* varied in placement in every analysis, but never occurred with other Tetracampidae. The two representatives of Mongolocampinae and Platynocheilinae were clustered in a monophyletic group in all analyses with very high support, and most likelihood results grouped them with Eunotini (Pteromalidae: Eunotinae; excluding *Idioporus*), however with low support.

#### Torymidae

Placement of Torymidae is uncertain, and it was proposed that the family arose from within the pteromalid lineage [121]. Historically, Torymidae have included Agaoninae and Sycophaginae ( = Idarninae), which were removed by Bouček [30]. Torymidae were revised by Grissell [34] and include only two subfamilies, the largely phytophagous Megastigminae and the mostly parasitic Toryminae, with the latter divided into seven tribes that encompassed the previously recognized Erimerinae, Monodontomerinae and Thaumatotoryminae and several taxa as *incertae sedis*. Campbell et al. [17] failed to find a monophyletic group, despite what they and Gibson et al. [9] noted to be strong morphological support for the family.

Torymidae were never monophyletic, but Megastigminae and Toryminae were each monophyletic with very strong support (Table 3). Support for tribes within Toryminae was variable. Torymini were monophyletic with low to very strong support in all analyses except parsimony, and Podagrionini were either monophyletic mostly with low support (62% of likelihood analyses) or paraphyletic. Monodontomerini were monophyletic with strong bootstrap support in all analyses, but with the inclusion of the unplaced *Zaglyptonotus* and exclusion of *Chrysochalcissa* which clusters deep within Microdontomerini. *Echthrodape* (Toryminae *incertae sedis*) was previously placed in Eucharitidae and Perilampidae and then Torymidae by Grissell [34]. This genus was recovered as the sister group of Microdontomerini. The unplaced *Glyphomerus* exemplars remained unplaced within Toryminae with no particular association with other tribes. The two representatives of Palachiini grouped either with Torymoidini or Podagrionini, but never together. None of the groups seemed to be impacted by the inclusion or exclusion or RAAs. No logical sister groups were identified for either subfamily.

#### Trichogrammatidae

Trichogrammatidae are well defined and according to Bouček and Noyes [116], are possibly the only monothetic family of Chalcidoidea. Owen et al. [35] assessed higher-level groups and generic relationships based on molecular and morphological evidence and recognized a paraphyletic Trichogrammatinae and monophyletic Oligositinae. Of the groups sampled herein, *Ceratogramma* (Trichogrammatinae; unplaced to tribe) were recognized as the sister group of the remaining Trichogrammatidae.

Trichogrammatidae were monophyletic in nearly all of our analyses (94% of the MJR consensus trees), but with low BS support in likelihood analyses only after the inclusion of RAAs. *Ceratogramma* was sister to the remaining Trichogrammatidae in all results, except for one analysis when it was excluded from the family (Table 3, SSNR). Our internal relationships mirror those of Owen et al. [35]. Trichogrammatidae were sister to Azotinae in all but the parsimony analysis, which placed them as a sister group of *Idioporus*, *Rotoita* and *Systolomorpha*.

### Conclusions

Is the diverse and unsupported backbone of Chalcidoidea the product of a rapid radiation event [48], [122]? Mymaridae first appear in the early to mid Cretaceous [6]. Based on what appear to be valid fossils of Eulophidae and Trichogrammatidae, there are records of higher-level chalcidoids in only one mid-Cretaceous deposit [8], with records of the same age other than Mymaridae more questionable [6]. The diversification of chalcidoid families does not appear until the Eocene, with modern genera common in Oligocene and Miocene amber deposits [6]. Chalcidoids are mostly parasitoids, and their host groups in the Hemiptera and Holometabola were all undergoing an explosive radiation during the same period at the end of the Cretaceous [123], and a similar tracking of host diversification is not unexpected.

Using an array of nuclear protein coding genes but with fewer taxa, Desjardins et al. [18] found similar results that showed a weak backbone of relationships across their chalcidoid groups sampled. Given a scenario of explosive radiation of Chalcidoidea during a relatively short time period, it may be difficult to resolve higher group relationships with confidence [122]. However, the trees that we have recovered can help to evaluate some scenarios within a context of which groups are consistently supported and their relationships on the various tree topologies. These molecular results provide a unique perspective for examining relationships and hypotheses of chalcidoid evolution, especially in a group prone to morphological convergence.

What is the ancestral mode of host association for Chalcidoidea? Bouček [124] proposed Cleonyminae or some other wood-beetle parasitoids as having the most ancestral forms, but hypothesized that phytophagy could be plesiomorphic for the superfamily. This latter assumption was based on his observation that phytophagous species tend to be primitive within their respective groups. The placement of Chalcidoidea as sister group to either Diaprioidea or Proctotrupoidea *sensu stricto* and the basal sister group placement of Mymaridae argue against Bouček's hypothesis of a phytophagous ancestor. As well, the phytophagous groups are scattered across the tree and almost never basal within a particular lineage, as in with gall-forming Opheliminae derived from within Eulophidae, or seed-feeding Megastigminae, which are distantly placed from their proposed sister group, the Toryminae (Fig. 9).

Noyes [15] argued for a monophyletic Mymaridae + (Rotoitidae + Tetracampidae) as the sister group of the remaining Chalcidoidea. Our results somewhat support his hypothesis, placing Mymaridae and Rotoitidae at the base of the chalcidoid tree (Fig. 1), but with a different phylogenetic ordering, and with Tetracampidae both polyphyletic and placed more distally on the various topologies. Morphological evidence supports a sister group relationship between Mymaridae and the remaining Chalcidoidea [10], [16], [61]. Our results and more comprehensive analyses of Hymenoptera [11], [13] strongly support this hypothesis. Likelihood results place Rotoitidae as the sister group of the remaining Chalcidoidea after Mymaridae.

Mymaridae are virtually all egg parasitoids, primarily of Auchenorrhyncha, Heteroptera and Coleoptera [125]. The only known exception is for two species of *Stethynium* attacking larvae of *Ophelimus* (Eulophidae) [126]. We included *S. ophelimi* in our analysis, and its derived placement within the family suggests a secondary derivation of larval parasitism (Fig. 1). Egg parasitism is likely the ancestral trait for Mymaridae. Within the remaining Chalcidoidea, egg parasitism occurs in all Trichogrammatidae and a few other scattered taxa (Fig. 9). None of our results placed these chalcidoid egg parasitoids close to the root of Chalcidoidea. Is it possible for egg parasitism to be ancestral for the superfamily? Mymarommatoidea may be egg parasitoids of Psocoptera [127]. The small body size of Rotoitidae suggests that they also might be egg parasitoids, but there is not even a suspected host for this group [9]. Diaprioidea are primarily larval parasitoids of fly larvae or pupae with a few taxa hyperparasitic on Dryinidae or Formicidae [128]; none are egg parasitoids. Even if Mymarommatoidea are resolved as the sister group of Chalcidoidea (only in some of our results), the biology of these and Rotoitidae will need to be resolved before we can confidently consider egg parasitism as a basal trait for the superfamily.

Associated with an extreme diversity of host use, larval morphology is extremely diverse in Chalcidoidea [129]. Two types of hypermetamorphic development occur in Hymenoptera [130]. Type II involves deposition away from the host of a sclerotized planidiform first-instar larva that transforms in later instars to a typical weakly sclerotized sac-like hymenopteriform larva. Within Hymenoptera, this occurs only in one genus of Ichneumonidae (*Euceros*) and in Perilampidae (including Philomidinae) and Eucharitidae [95]. Although not recovered across all analyses, our results offer support for the single development of this trait within Chalcidoidea (Fig. 9).

Another important trait is the use of sessile Sternorrhyncha as hosts within Chalcidoidea, which ultimately leads to their importance in biological control programs. Mapping sternorrhynchan parasitism, either as primary parasitoids or hyperparasitoids, onto our current ‘best’ hypothesis shows a general scattering of host use that suggests multiple independent host shifts to this group. Probably most significant is the lack of grouping in any of our analyses of Encyrtidae and the aphelinid subfamilies Aphelininae, Azotinae, Coccophaginae, Eretmocerinae and Euryischiinae, which have in the past been treated as a single family [66]. Our results suggest that any traits associated with successful host use of Sternorrhyncha are independent events, and especially within Aphelinidae, should not be considered as phylogenetically linked. This is also important when we consider the single origin of heteronomy, or alternate host use by different sexes, which occurs only in the monophyletic Coccophaginae (Fig. 9).

Our results present the most comprehensive phylogenetic analysis of relationships Chalcidoidea based only on molecular data.. While not robust across the backbone of relationships within Chalcidoidea, they offer some firm insights into the origin and evolution of this important and highly diverse group of insects. Monophyly of many of the traditional groups is supported, and the secondary structure alignment and data set will be useful for future studies. Many changes in the higher classification of taxa within Chalcidoidea are suggested by these results. However, we reserve any judgment on these changes until our combined morphological and molecular analyses are complete.

## Supporting Information

Figure S1 Parsimony analysis of SSME dataset using TNT (31,607 steps; r.i. 0.62, strict consensus of >10,000 trees). Bootstrap values plotted to nodes with values greater than 95% represented by dot.
(PDF)

Table S1 Specimens sequenced and deposition information for specimen data and genebank accession numbers.
(XLS)

Nexus File S1 Chalcidoidea SSME dataset.
(NEX)
