# Supplementary figures and images for: A Molecular Phylogeny of the Chalcidoidea (Hymenoptera)
Source: PLoS One. 2011 Nov 3;6(11):e27023. doi: 10.1371/journal.pone.0027023 (PMC3207832; doi:10.1371/journal.pone.0027023)

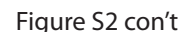

Supplement: Figure S1 — Parsimony analysis of SSME dataset using TNT (31,607 steps; r.i. 0.62, strict consensus of >10,000 trees). Bootstrap values plotted to nodes with values greater than 95% represented by dot. (PDF) [file pone.0027023.s001.pdf]
